# Proteomic associations with forced expiratory volume: a Mendelian randomisation study

**DOI:** 10.1186/s12931-023-02587-z

**Published:** 2024-01-18

**Authors:** Gisli Thor Axelsson, Thorarinn Jonmundsson, Youngjae Woo, Elisabet Alexandra Frick, Thor Aspelund, Joseph J. Loureiro, Anthony P. Orth, Lori L. Jennings, Gunnar Gudmundsson, Valur Emilsson, Valborg Gudmundsdottir, Vilmundur Gudnason

**Affiliations:** 1 Icelandic Heart Association, Holtasmari 1, 201 Kopavogur, Iceland; 2 Department of Internal Medicine, Landspitali University Hospital, 101 Reykjavik, Iceland; 3 Novartis Biomedical Research, Cambridge, MA 02139 USA; 4 Faculty of Medicine, University of Iceland, 101 Reykjavik, Iceland; 5 Novartis Institutes for Biomedical Research, San Diego, CA 92121 USA; 6 Department of Respiratory Medicine and Sleep, Landspitali University Hospital, 108 Reykjavik, Iceland

**Keywords:** Lung function tests, Forced expiratory volume, Proteomics, Mendelian randomisation

## Abstract

**Background:**

A decline in forced expiratory volume (FEV1) is a hallmark of respiratory diseases that are an important cause of morbidity among the elderly. While some data exist on biomarkers that are related to FEV1, we sought to do a systematic analysis of causal relations of biomarkers with FEV1.

**Methods:**

Data from the population-based AGES-Reykjavik study were used. Serum proteomic measurements were done using 4782 DNA aptamers (SOMAmers). Data from 1479 participants with spirometric data were used to assess the association of SOMAmer measurements with FEV1 using linear regression. Bi-directional two-sample Mendelian randomisation (MR) analyses were done to assess causal relations of observationally associated SOMAmers with FEV1, using genotype and SOMAmer data from 5368 AGES-Reykjavik participants and genetic associations with FEV1 from a publicly available GWAS (n = 400,102).

**Results:**

In observational analyses, 530 SOMAmers were associated with FEV1 after multiple testing adjustment (FDR < 0.05). The most significant were Retinoic Acid Receptor Responder 2 (RARRES2), R-Spondin 4 (RSPO4) and Alkaline Phosphatase, Placental Like 2 (ALPPL2). Of the 257 SOMAmers with genetic instruments available, eight were associated with FEV1 in MR analyses. Three were directionally consistent with the observational estimate, Thrombospondin 2 (THBS2), Endoplasmic Reticulum Oxidoreductase 1 Beta (ERO1B) and Apolipoprotein M (APOM). THBS2 was further supported by a colocalization analysis. Analyses in the reverse direction, testing whether changes in SOMAmer levels were caused by changes in FEV1, were performed but no significant associations were found after multiple testing adjustments.

**Conclusions:**

In summary, this large scale proteogenomic analyses of FEV1 reveals circulating protein markers of FEV1, as well as several proteins with potential causality to lung function.

**Supplementary Information:**

The online version contains supplementary material available at 10.1186/s12931-023-02587-z.

## Background

Chronic respiratory diseases such as chronic obstructive pulmonary disease (COPD) are a leading global cause of mortality and morbidity, with their relative importance increasing in the last decades [1]. Diagnosis of COPD is based on pulmonary function testing, by a low forced expiratory volume in one second (FEV1) relative to the forced vital capacity (FVC), and a progressive decline in pulmonary function is a feature of the disease [2]. Therefore, a decline in FEV1 is a hallmark of respiratory diseases that bring a great burden of disease to individuals and societies. While it is undisputed that exposure to external harmful stimuli such as cigarette smoke and biomass fumes are substantial risk factors for pulmonary function decline, intrinsic factors such as genetics and gene-environment interactions play a significant part as well [2–4]. Genome wide association studies (GWAS) have found several genetic polymorphisms that are associated with COPD or lung function decline, including polymorphisms in or near genes encoding matrix metalloproteinase 12, nicotinic acetylcholine receptor, hedgehog interacting protein, glutathione S-transferase, C-terminal domain–containing protein [5–9] and the antiprotease alpha-1-antitrypsin [10]. In addition to genetic polymorphisms, biomarkers that predict COPD or lung function have been discovered. Among them are inflammatory markers, soluble receptor for advanced glycoprotein end products (AGER), club cell secretory protein 16 (SCGB1A1) and surfactant protein D (SFTPD) [11–14]. For several of these biomarkers, estimations of potential causality have been made. AGER, SCGB1A1 and SFTPD have been suggested to be causally associated with COPD or with lung function, while analyses have pointed against such an association for the inflammatory markers CRP and IL-6 [15–19]. Still, while substantial epidemiologic data exist regarding single genetic markers and biomarkers that predict lung function or diseases whose diagnosis is based on impaired lung function, a systematic large-scale analysis of observational and causal associations of protein markers with lung function has not been undertaken to our knowledge.

Proteomics have emerged as a way of exploring molecular signatures of disease, especially with the advent of methods that allow for measurement and evaluation of thousands of proteins in biological samples from participants of large cohort studies [20]. In addition to predicting disease and disease-related outcomes, integration of genetic data allows one to make assumptions regarding the causality of proteomic markers. Mendelian randomization (MR) is such a method and utilizes genetic polymorphisms as instrumental variables to assess the relationship of an exposure with an outcome. As chromosomal alleles are randomly allocated during gamete formation, this methodology allows one to avoid the effect of confounders and to infer causality from epidemiologic data [21].

The aim of the study was to systematically explore the associations of a multitude of protein markers with lung function in an elderly population, focusing on FEV1 as the main outcome. Then, the aim was to assess the potential causal relationships of these protein markers with FEV1 by use of bi-directional two-sample Mendelian randomization.

## Methods

### Study phenotyping

The Age/Gene Environment Susceptibility (AGES)-Reykjavik study is a population-based cohort study of 5,764 elderly Icelanders that was carried out between 2002 and 2006. The participants, aged from 66 to 96 years (mean 76 years), were all prior participants of the Reykjavik Study done decades earlier. As part of AGES-Reykjavik, the participants underwent extensive phenotyping by questionnaires, physiological measurements, imaging studies and laboratory measurements, during a three-day period. The study was approved by the Icelandic National Bioethics Committee (VSN-00-063), in accordance with the Helsinki Declaration and the Institutional Review Board of the Intramural Program of the National Institute for Aging, with informed consent obtained from all participants. Further details of the study design are previously published [22].

A random subset of the study participants underwent lung function testing in a standardised manner. The device used was a Vitalograph Gold Standard Plus (Vitalograph Ltd., UK). Each participant completed three attempts. Participants with spirometry of acceptable quality were included. Spirometry measures from participants that completed at least two attempts with a no more than 300 ml difference between the attempts and exhalation for at least 6 s were deemed acceptable [23]. Smoking history was ascertained from questionnaires while anthropometric measurements were done during the clinic visit. Protein measurements were done in serum samples from participants using a high throughput proteomics technology, the SOMAscan (SomaLogic, Boulder, CO) in which DNA aptamers (Slow-Off Rate Modified Aptamers (SOMAmers)) bind to target protein epitopes and are then quantified with the help of fluorescence after wash-out of unbound aptamers and proteins. Measurements in AGES-Reykjavik were done with a 5034 SOMAmer platform in serum from 5457 AGES-Reykjavik participants. For the analyses, 4782 SOMAmers targeting 4135 human proteins were used, excluding SOMAmers annotated to non-human proteins. Measurement data were transformed using Box-Cox transformation and extreme outliers were excluded, as previously described [24].

### Statistical analyses

**Fig. 1:**
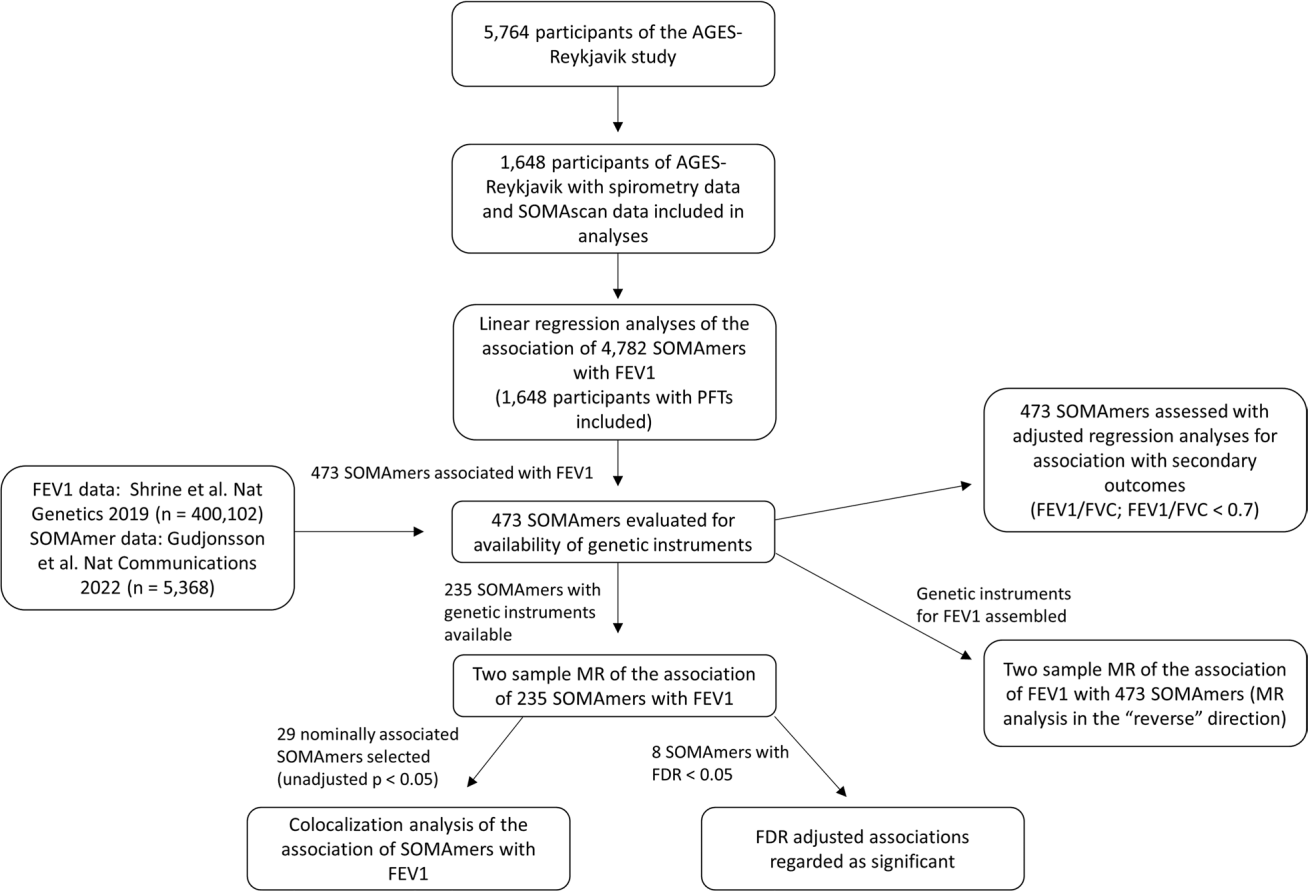
A flow chart of the study design

A flow chart of study design is shown in Fig. 1. Descriptive statistics were compiled for participants with acceptable pulmonary function tests, demographic covariate data and protein measurements available (n = 1479). Using data from these participants, the association of all measured human SOMAmers (n = 4782) with FEV1 was assessed using linear regression modelling. These analyses were adjusted for variables that are commonly used to predict FEV1 in clinical practice: age, sex, height, age squared, and height squared [25]. Adjustment for multiple testing was done using a Benjamini–Hochberg False Discovery Rate (FDR), where FDR < 0.05 was considered statistically significant. To understand how history of tobacco smoking, the most important lifestyle factor influencing lung function, affected these associations, the analyses were repeated with participants stratified by smoking history (ever-smokers versus never-smokers). In a secondary analysis, all SOMAmers associated (FDR < 0.05) with FEV1 were tested for association with FEV1/FVC ratio and a FEV1/FVC ratio under 0.7, a value used in the clinical diagnosis of COPD [2], using a linear and logistic regression, respectively. These analyses were adjusted for the same covariates as the primary analysis for FEV1. Genes encoding proteins significantly associated with FEV1 after adjustment for multiple testing (FDR < 0.05) were subjected to over-representation analysis of Gene Ontology terms [26].

For SOMAmers that were associated with FEV1 after adjustment for multiple testing (FDR < 0.05), a bi-directional two-sample MR analysis was performed to assess support for causal relationships between SOMAmers and FEV1, in either direction. When testing the causal effect of SOMAmer on FEV1 (forward direction), the MR analysis was restricted to SOMAmers with genetic instruments available, which were defined as follows. The associations of single-nucleotide polymorphisms (SNPs) with SOMAmers were calculated using data from 5,368 participants of AGES-Reykjavik, as previously published and described in detail [24]. The possible instrumental variables for each SOMAmer were defined as SNPs located within a cis-window for the gene coding for the protein measured by the SOMAmer, defined as within 500 kb up- and downstream of the gene. SNPs with a window-wide significant association (p < 0.05/number of SNPs in cis-window) with a given SOMAmer were considered as potential instruments. For each gene, SNPs were filtered based on linkage disequilibrium (LD; r^2^ < 0.2) or distance (> 1 mb) using Plink v1.9. As the forward MR analysis was restricted to a single region on the genome, a more inclusive LD threshold compared to the reverse MR was chosen to increase statistical power [27]. Instruments were considered valid if F > 10 for the association of instruments with SOMAmers [28]. The associations of the instrumental variables with FEV1 were obtained from a GWAS of lung function [29] in which data from two cohorts, UKBiobank and SpiroMeta Consortium, were meta-analysed. The total number of participants in that analysis was 400,102 [29]. A MR analysis in the reverse direction to assess the causal effect of FEV1 on SOMAmer levels was performed using all SOMAmers associated with FEV1 in the observational linear regression model (FDR < 0.05) as outcomes. Genetic instruments for FEV1 were selected based on the same GWAS of lung function as described above [29]. For these analyses, SNPs associated with FEV1 (p < 5 × 10^–8^) were defined as instruments after clumping by an LD threshold of r^2^ < 0.01. The associations of the instrumental variables with SOMAmer levels were obtained from AGES-Reykjavik results [24]. For the MR analyses, SNP reference alleles were harmonised between studies using the TwoSampleMR R package [30]. The MR estimates for FEV1 were obtained using the generalized weighted least squares (GWLS) method [31], which accounts for correlation between instruments, except for instances where only one instrument was available, in which Wald ratios were calculated. For each MR analysis with more than two instruments, sensitivity analyses using the weighted median and Egger estimators were done to assess the validity of instruments and limit the effect of pleiotropic associations, respectively. Results were considered to pass these sensitivity analyses when the following conditions were met. For the weighted median estimator, the weighted median estimate had to be significant and directionally consistent with the GWLS estimate and for Egger, the Egger estimate had to be directionally consistent with the GWLS estimate and the intercept not significant. Additionally, for SOMAmers associated with FEV1 in the forward MR analysis, a leave-one-out analysis was performed. For analyses in the reverse direction, estimates were obtained using the inverse variance weighted method. Adjustment for multiple testing was done using the Benjamini–Hochberg False Discovery Rate (FDR).

A colocalization analysis was performed to provide additional causal support for analytes associated with FEV1 in MR analyses. Here, AGES-Reykjavik serum protein quantitative trait loci (pQTLs) [24], summary statistics for FEV1 [29] and plasma pQTLs from a cohort study of over 35,000 Icelanders designed to associate genetics, proteins and disease, published by deCODE Genetics [32] were harmonized to account for strand orientation and differences in genome builds. All studies were lifted over to build GRCh38 for colocalization when needed. For the QTLs, only putative cis-regulatory variants were examined defined by 500kb away from the gene body. All regions with variants that had associations of P < 1 × 10^–5^ were fine-mapped using Sum of Single Effects (SuSiE) [33] based on 1000 genome population reference [34]. 95% credible-sets were filtered and colocalization between GWAS and QTLs was performed using fastENLOC using the posterior inclusion probabilities estimated in SuSiE [33, 35]. Regional colocalization probability (RCP) was calculated by summing the colocalization probability within each 95% credible-set and filtered colocalization results at RCP > 80% for further examination. RCP represents the probability that a given genomic region contains a single colocalized variant [36]. Visualization was done using Julia 1.7 and libraries within [37].

## Results

### The protein profile of pulmonary function

Descriptive statistics for participants that had pulmonary function testing data are shown in Table 1. A majority of participants were female (54%) and participants were on average 76 years old. Most participants had a history of smoking (60%) while a minority had evidence of obstruction on spirometry (37%).

**Table 1:** Overview of study participants

n	1479
Sex = Female (%)	804 (54.4)
Age—years [mean (SD)]	76.4 (5.8)
Height—cm [mean (SD)]	167.4 (9.5)
Weight—kg [mean (SD)]	75.5 (14.7)
BMI—kg/m^2^ [mean (SD)]	26.8 (4.3)
Eversmoker (%)†	863 (59.9)
FEV1—L [mean (SD)]	2.13 (0.69)
FEV1—% of predicted* [mean (SD)]	0.89 (0.22)
FVC—L [mean (SD)]	2.99 (0.84)
FVC—% of predicted* [mean (SD)]	0.92 (0.17)
Low (70%) FEV1/FVC (%)	548 (37.1)

^†^38 participants had missing smoking data

*Predicted values based on Hankinson et al. [25]

Summary statistics for all AGES-Reykjavik participants with genotype data available are previously published [24]

BMI: Body Mass Index

FEV1: Forced expiratory volume in the first second

FVC: Forced vital capacity

SD: Standard deviation

L: Litres

**Fig. 2:**
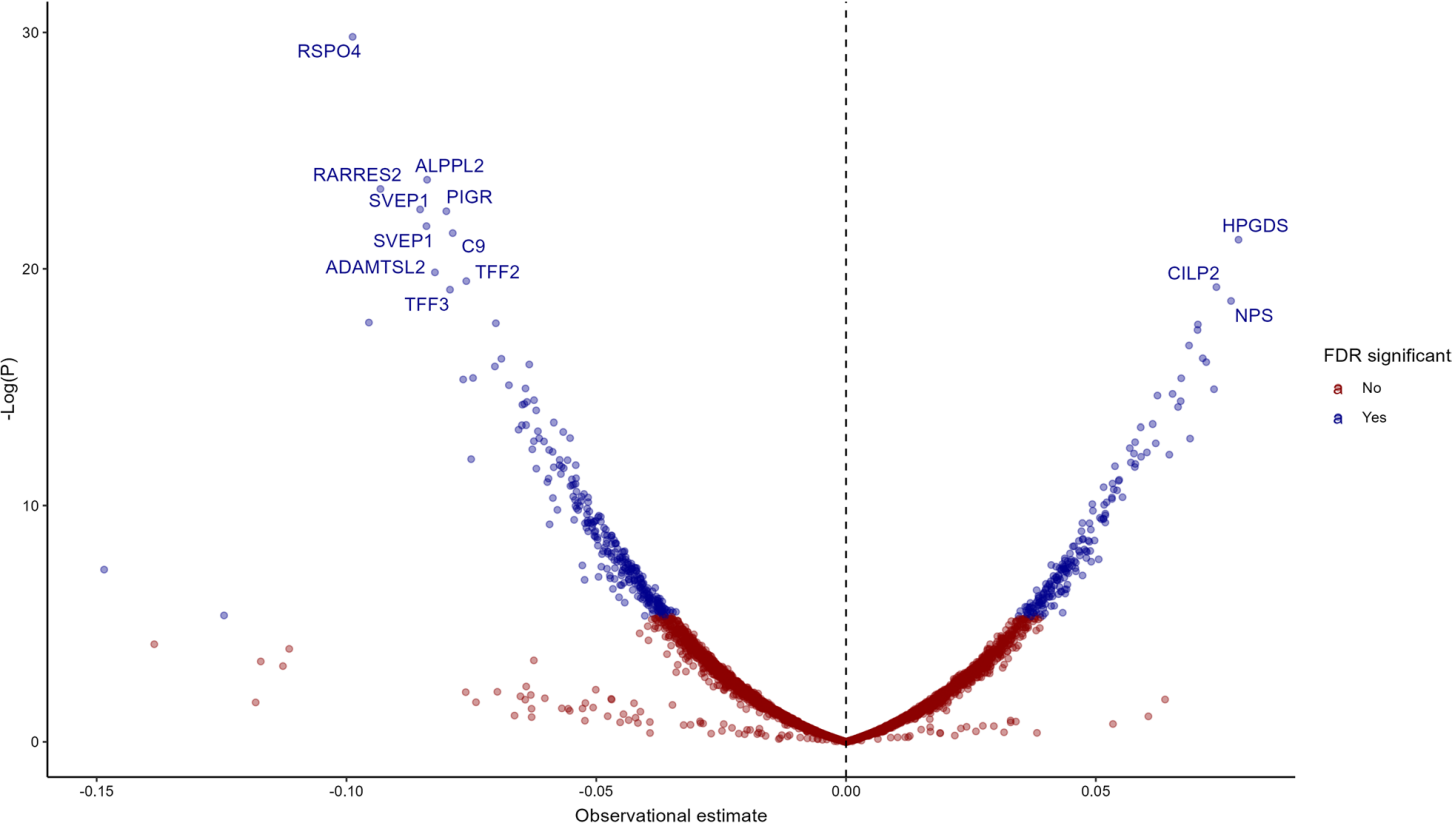
A volcano plot showing the observational associations of all 4782 SOMAmers with FEV1

Of the 4,782 SOMAmers tested, 530 were observationally associated (FDR < 0.05) with FEV1 after adjustment for multiple testing (Table 2, Additional file 5: Table S1, Fig. 2). The most significantly associated SOMAmers measured were Retinoic Acid Receptor Responder 2 (RARRES2, β = − 0.103, p = 7.76 × 10^–12^), R-Spondin 4 (RSPO4, β = − 0.094, p = 1.86 × 10^–11^), Alkaline Phosphatase, Placental Like 2 (ALPPL2, β = − 0.087, p = 1.23 × 10^–10^), Complement C9 (C9, β = − 0.084, p = 3.04 × 10^–10^) and Hematopoietic Prostaglandin D Synthase (HPGDS, β = 0.083, p = 5.12 × 10^–10^). Results for proteins that have been previously suggested as biomarkers of FEV1 [11, 13] are shown in Additional file 6: Table S2. Prior associations of SCGB1A1 (β = − 0.034, p = 0.02), SFTPD (β = − 0.041, p = 1.67 × 10^–3^), CRP (β = − 0.065, p = 2.72 × 10^–7^), fibrinogen (β = − 0.041, p = 2.8 × 10^–3^ for the stronger associated SOMAmer), IL6 (β = − 0.054, p = 1.5 × 10^–4^ for the stronger associated SOMAmer), eotaxin (β = − 0.051, p = 1.1 × 10^–4^) and TNF (β = − 0.028, p = 0.04 for the stronger associated SOMAmer) were reproduced in the AGES-Reykjavik data while associations for other suggested biomarkers of FEV1 were not.

**Table 2:** Observational associations of the 25 proteins with the most significant associations for FEV1 as selected by the lowest p-values

SOMA	EGS	β	95% CI	p	FDR
3079_62_2	RARRES2	− 0.103	− 0.132 to − 0.074	7.76 × 10^–12^	3.71 × 10^–8^
8464_31_3	RSPO4	− 0.094	− 0.122 to − 0.067	1.86 × 10^–11^	4.44 × 10^–8^
7813_6_3	ALPPL2	− 0.087	− 0.113 to − 0.061	1.23 × 10^–10^	1.96 × 10^–7^
2292_17_4	C9	− 0.084	− 0.109 to − 0.058	3.04 × 10^–10^	3.63 × 10^–7^
12549_33_3	HPGDS	0.083	0.057 to 0.109	5.12 × 10^–10^	4.37 × 10^–7^
6605_17_3	IGFALS	0.083	0.057 to 0.109	5.99 × 10^–10^	4.37 × 10^–7^
12707_26_3	DPYSL3	0.083	0.057 to 0.109	6.40 × 10^–10^	4.37 × 10^–7^
9191_8_3	TFF2	− 0.084	− 0.111 to − 0.058	7.42 × 10^–10^	4.44 × 10^–7^
8841_65_3	CILP2	0.082	0.056 to 0.108	1.09 × 10^–9^	5.78 × 10^–7^
6379_62_3	ADAMTSL2	− 0.088	− 0.116 to − 0.059	3.18 × 10^–9^	1.52 × 10^–6^
11178_21_3	SVEP1	− 0.082	− 0.11 to − 0.054	1.03 × 10^–8^	4.46 × 10^–6^
2677_1_1	EGFR	0.075	0.049 to 0.1	1.21 × 10^–8^	4.56 × 10^–6^
8885_6_3	CACNA2D3	0.077	0.051 to 0.104	1.24 × 10^–8^	4.56 × 10^–6^
11109_56_3	SVEP1	− 0.081	− 0.109 to − 0.053	1.88 × 10^–8^	6.44 × 10^–6^
13722_105_3	C9	− 0.073	− 0.099 to − 0.048	2.23 × 10^–8^	7.07 × 10^–6^
10514_5_3	PTGDS	− 0.086	− 0.116 to − 0.056	2.37 × 10^–8^	7.07 × 10^–6^
2658_27_1	NTRK3	0.075	0.048 to 0.101	3.67 × 10^–8^	1.03 × 10^–5^
6390_18_3	NPS	0.078	0.05 to 0.106	4.61 × 10^–8^	1.22 × 10^–5^
6075_61_3	HEXB	0.076	0.049 to 0.103	6.78 × 10^–8^	1.71 × 10^–5^
3396_54_2	REN	− 0.075	− 0.102 to − 0.048	7.42 × 10^–8^	1.77 × 10^–5^
8323_163_3	TFF3	− 0.076	− 0.104 to − 0.048	9.06 × 10^–8^	2.06 × 10^–5^
2609_59_2	CST3	− 0.081	− 0.111 to − 0.051	1.14 × 10^–7^	2.49 × 10^–5^
3216_2_2	PIGR	− 0.07	− 0.096 to − 0.044	1.35 × 10^–7^	2.81 × 10^–5^
4496_60_2	MMP12	− 0.072	− 0.098 to − 0.045	1.52 × 10^–7^	3.03 × 10^–5^
5632_6_3	CRTAC1	0.074	0.046 to 0.102	1.93 × 10^–7^	3.69 × 10^–5^

Results of linear regression analyses adjusted for sex, age, age squared, height and height squared

SOMA: SOMAmer number

EGS: Entrez Gene symbol

95% CI: 95% Confidence interval

p: p-value

FDR: False Discovery Rate (adjusted p-value)

The proteins most significantly associated with FEV1 overall also had strong associations with FEV1 among ever-smokers, such as RARRES2 (β = − 0.12, p = 5.85 × 10^–9^), RSPO4 (β = − 0.095, p = 9.61 × 10^–7^), ALPPL2 (β = − 0.097, p = 1.05 × 10^–7^) and Complement 9 (C9, β = − 0.096, p = 2.05 × 10^–7^). Trefoil Factor 2 (TFF2, β = − 0.104, p = 3.37 × 10^–8^), and Neurotrophic Receptor Tyrosine Kinase 3 (NTRK3, β = 0.107, p = 2.47 × 10^–8^) also had notably strong associations with the outcome. However, associations of these proteins were much weaker among never-smokers, with the strongest associations observed for two SOMAmers measuring SVEP1 (β = − 0.082, p = 1.64 × 10^–5^ for the stronger associated SOMAmer) (Additional file 6: Table S3) in this subgroup. Of the 530 proteins associated with FEV1, 224 were also associated (FDR < 0.05) with FEV1/FVC (42%) and 160 (30%) were associated with an obstructive deficit on spirometry (FEV1/FVC < 0.7; Additional file 1: Figure S1, Additional file 5: Table S1). Among the 25 proteins most strongly associated with FEV1, including RARRES2, RSPO4 and ALPPL2, 19 were associated with continuous FEV1/FVC and 16 were associated with FEV1/FVC under 0.7 (Additional file 6: Table S4). The 530 proteins associated with FEV1 in linear regression analyses were most strongly enriched for Gene Ontology terms related to compliment activation, extracellular matrix organization, peptidase regulator activity, humoral immune response and regulation of neurogenesis (Additional file 2: Figure S2, Additional file 5: Table S5).

### Mendelian randomization analysis

**Fig. 3:**
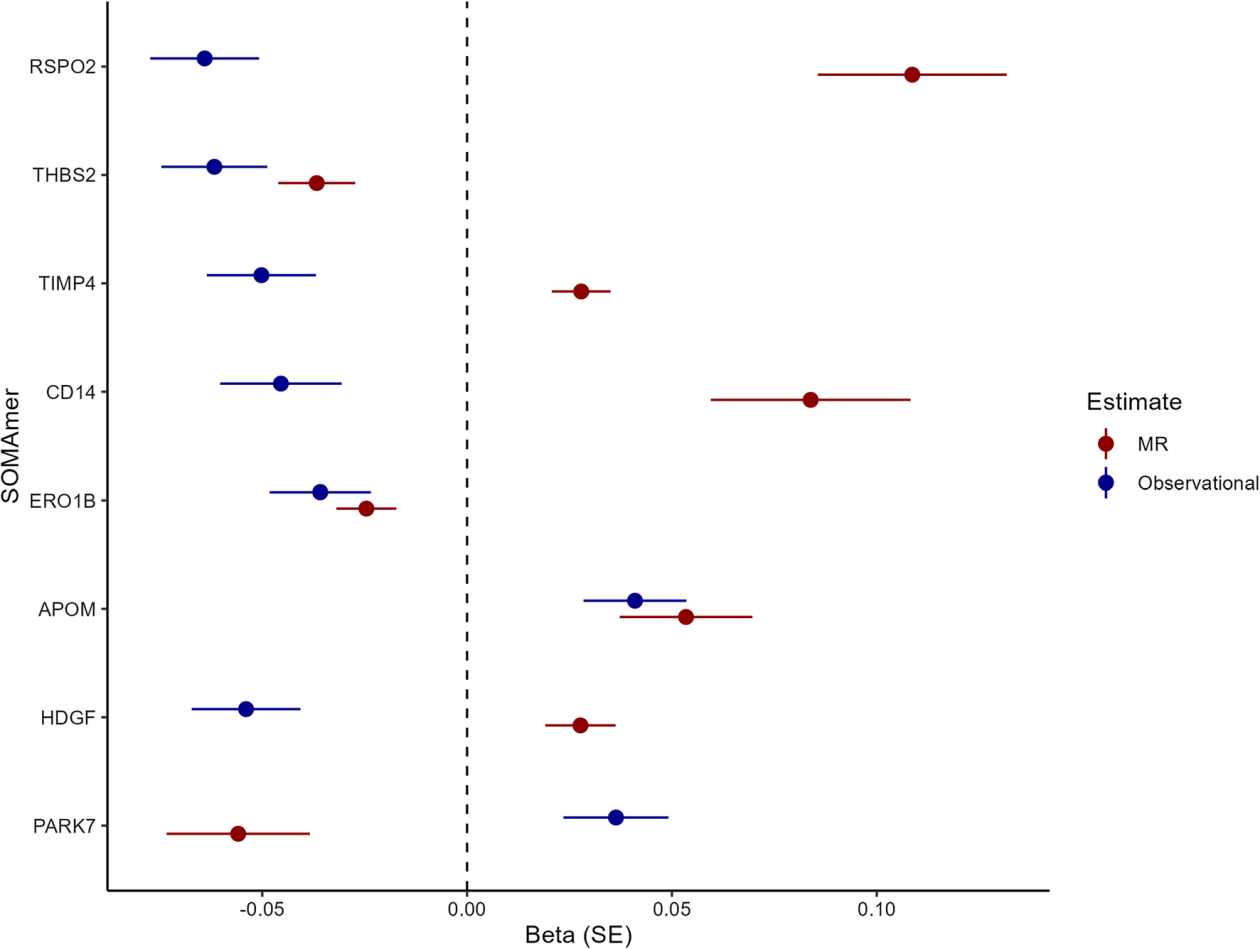
A forest plot showing the observational and Mendelian randomisation estimates for FEV1 for causally associated SOMAmers (FDR < 0.05 for the weighted median estimate of the association of the SOMAmer with FEV1)

Of the 530 SOMAmers observationally associated with FEV1, 257 (49%) had genetic instruments available (Additional file 5: Table S1) and were included in MR analyses to evaluate potentially causal associations of SOMAmers with FEV1. The instruments are shown in Additional file 5: Table S6 for SOMAmers with nominally significant (unadjusted p < 0.05) MR associations. Table 3 shows the 35 SOMAmers that were nominally associated with FEV1 (unadjusted p < 0.05) in the MR analysis and passed weighted median sensitivity testing. Eight were significantly associated with FEV1 in the MR analysis (FDR for MR estimate < 0.05; Table 3), suggesting they may have a causal effect on lung function. Of the seven associations based on more than two genetic instruments, none were solely driven by a single variant (Additional file 4: Figure S4). Three SOMAmers that were significant (FDR < 0.05) in the MR analysis, Thrombospondin 2 (THBS2, β = − 0.037, p = 9.53 × 10^–5^), Endoplasmic Reticulum Oxidoreductase 1 Beta (ERO1B, β = − 0.025, p = 8.05 × 10^–4^) and Apolipoprotein M (APOM, β = 0.053, p = 9.72 × 10^–4^) were directionally consistent with the observational analyses. The other five SOMAmers with significant causal estimates, R-Spondin-2 (RSPO2), TIMP Metallopeptidase Inhibitor 4 (TIMP4), interleukin 1 receptor antagonist (IL1RN), CD14 and Heparin Binding Growth Factor (HDGF) were directionally inconsistent (Fig. 3). Among all 35 nominally associated SOMAmers, the directional consistency between causal and observational estimates was low, or 34%, suggesting that the potentially causal effects of the proteins are generally not reflected in the observational estimates. However, these nominally associated SOMAmers included SERPINA1, which measures the level of alpha-1-antitrypsin, deficiency of which is a well-established causal determinant of impaired lung function via emphysema formation [38] (Table 3). Of the five previously suggested biomarkers of FEV1 listed in Additional file 6: Table S2 that were associated with FEV1 in linear regression analyses (FDR < 0.05), only three proteins had genetic instruments available, the acute phase reactants CRP and fibrinogen as well as eotaxin. None of these proteins were associated with FEV1 in the MR analysis (Additional file 6: Table S7).

**Table 3:** Results of Mendelian randomisation analysis for the association of SOMAmers with FEV1

SOMA	Protein	nSNP	Β—MR	SE	P—MR	FDR P—MR	Egger	Β—Obs	P—Obs	FDR P—Obs
**8409_3_3**	**RSPO2**	**3**	**0.109**	**0.023**	**2.44 × 10**^**–6**^	**6.27 × 10**^**–4**^	**Yes**	− **0.071**	**6.13 × 10**^**–7**^	**9.17 × 10**^**–5**^
***3339_33_1***	***THBS2***	***24***	− ***0.037***	***0.009***	***9.53* × *10***^***–5***^	***8.58* × *10***^***–3***^	***Yes***	− ***0.054***	***9.87* × *10***^***–5***^	***3.44* × *10***^***–3***^
**6462_12_3**	**TIMP4**	**17**	**0.028**	**0.007**	**1.00 × 10**^**–4**^	**8.58 × 10**^**–3**^	**Yes**	− **0.048**	**6.78 × 10**^**–4**^	**0.012**
**5353_89_2**	**IL1RN**	**3**	**0.067**	**0.018**	**1.44 × 10**^**–4**^	**9.26 × 10**^**–3**^	**Yes**	− **0.04**	**3.56 × 10**^**–3**^	**0.038**
**8969_49_3**	**CD14**	**6**	**0.084**	**0.024**	**5.85 × 10**^**–4**^	**0.03**	**Yes**	− **0.055**	**4.96 × 10**^**–4**^	**0.01**
***7994_41_3***	***ERO1B***	***12***	− ***0.025***	***0.007***	***8.05* × *10***^***–4***^	***0.034***	***Yes***	− ***0.038***	***4.28* × *10***^***–3***^	***0.042***
***14125_5_3***	***APOM***	***1***	***0.053***	***0.016***	***9.72* × *10***^***–4***^	***0.036***	***–***	***0.053***	***6.65* × *10***^***–5***^	***2.66* × *10***^***–3***^
**8953_47_3**	**HDGF**	**7**	**0.028**	**0.009**	**1.26 × 10**^**–3**^	**0.04**	**Yes**	− **0.046**	**8.38 × 10**^**–4**^	**0.014**
2668_70_2	CAPN1	3	− 0.051	0.016	1.94 × 10^–3^	0.055	Yes	0.049	6.91 × 10^–4^	0.013
8300_82_3	PEX14	1	− 0.086	0.029	2.70 × 10^–3^	0.069	–	0.043	1.98 × 10^–3^	0.025
*2828_82_2*	*SPINT1*	*3*	*0.04*	*0.014*	*4.73* × *10*^*–3*^	*0.102*	*Yes*	*0.041*	*3.66* × *10*^*–3*^	*0.038*
3290_50_2	CD109	49	− 0.013	0.005	4.91 × 10^–3^	0.102	Yes	0.051	8.83 × 10^–5^	3.28 × 10^–3^
2797_56_2	APOB	9	− 0.029	0.011	5.18 × 10^–3^	0.102	Yes	0.067	7.42 × 10^–7^	1.04 × 10^–4^
*3580_25_8*	*SERPINA1*	*9*	− *0.027*	*0.01*	*6.03* × *10*^*–3*^	*0.111*	*Yes*	− *0.06*	*1.68* × *10*^*–5*^	*9.77* × *10*^*–4*^
13123_3_3	FLRT3	59	− 0.008	0.003	8.97 × 10^–3^	0.149	Yes	0.044	1.31 × 10^–3^	0.019
*12395_86_3*	*DARS2*	*2*	− *0.058*	*0.022*	*9.64* × *10*^*–3*^	*0.149*	*–*	− *0.038*	*4.74* × *10*^*–3*^	*0.046*
*5939_42_3*	*TNFSF12*	*77*	*0.011*	*0.004*	*0.01*	*0.149*	*Yes*	*0.042*	*1.54* × *10*^*–3*^	*0.021*
8427_118_3	RSPO3	17	0.033	0.013	0.01	0.149	Yes	− 0.061	3.23 × 10^–5^	1.63 × 10^–3^
*2630_12_2*	*IL1RAP*	*58*	*0.006*	*0.002*	*0.018*	*0.228*	*No*	*0.043*	*1.05* × *10*^*–3*^	*0.016*
7822_11_3	HRASLS2	1	0.071	0.03	0.018	0.228	*–*	− 0.042	1.70 × 10^–3^	0.022
5070_76_3	TNFRSF6B	3	0.174	0.075	0.02	0.25	Yes	− 0.052	6.54 × 10^–5^	2.66 × 10^–3^
8992_1_3	TMEM2	1	− 0.052	0.023	0.025	0.282	*–*	0.038	4.33 × 10^–3^	0.042
8288_27_3	APOH	5	− 0.032	0.014	0.025	0.282	Yes	0.05	2.86 × 10^–4^	7.12 × 10^–3^
7803_4_3	CHST1	3	0.035	0.016	0.028	0.304	Yes	− 0.04	3.77 × 10^–3^	0.039
*13130_150_3*	*HK2*	*2*	− *0.04*	*0.019*	*0.032*	*0.304*	*–*	− *0.039*	*3.79* × *10*^*–3*^	*0.039*
9870_17_3	WARS	5	− 0.021	0.01	0.032	0.304	Yes	0.048	3.23 × 10^–4^	7.64 × 10^–3^
*4297_62_3*	*SPON1*	*8*	− *0.026*	*0.012*	*0.032*	*0.304*	*Yes*	− *0.046*	*1.39* × *10*^*–3*^	*0.019*
2789_26_2	MMP7	8	0.02	0.009	0.033	0.304	Yes	− 0.056	4.59 × 10^–5^	2.13 × 10^–3^
*9172_69_3*	*MMP8*	*13*	− *0.014*	*0.007*	*0.036*	*0.316*	*Yes*	− *0.041*	*2.16* × *10*^*–3*^	*0.026*
5646_20_3	RNASE6	33	0.008	0.004	0.037	0.316	Yes	− 0.055	5.32 × 10^–5^	2.29 × 10^–3^
*4546_27_3*	*ADGRE2*	*29*	*0.012*	*0.006*	*0.039*	*0.317*	*Yes*	*0.065*	*1.05* × *10*^*–6*^	*1.29* × *10*^*–4*^
3449_58_2	SERPINA4	56	− 0.007	0.004	0.039	0.317	Yes	0.038	4.68 × 10^–3^	0.045
10746_24_3	DKK3	6	− 0.031	0.015	0.044	0.327	Yes	0.063	5.72 × 10^–6^	4.66 × 10^–4^
12387_7_3	PDLIM4	11	− 0.025	0.012	0.044	0.327	Yes	0.049	5.60 × 10^–4^	0.011
5007_1_1	MAPK14	2	− 0.048	0.024	0.048	0.327	–	0.042	1.98 × 10^–3^	0.025

Shown are data for SOMAmers that had nominally significant (unadjusted p < 0.05) associations using Mendelian randomisation and passed weighted median sensitivity analyses. In the column with results from Egger analyses, “Yes” denotes passing the sensitivity test, “No” denotes failing and “–” denotes insufficient number of SNPs to perform the test. Observational data are adjusted for sex, age, age squared, height and height squared

**Bold** = Significantly associated with FEV1 in MR analyses after multiple testing correction (FDR < 0.05)

*Italic* = Consistent in direction between MR analyses and observational analyses

B—MR: Weighted median estimate from the mendelian randomization analysis of the association of the SOMAmer with FEV1

SE: Standard error for the weighted median estimate from the Mendelian randomization analysis of the association of the SOMAmer with FEV1

P—MR: P-value for the weighted median estimate from the Mendelian randomization analysis of the association of the SOMAmer with FEV1

FDR P—MR: False Discovery Rate adjusted p-value for the weighted median estimate from the Mendelian randomization analysis of the association of the SOMAmer with FEV1

B—Obs: Beta estimate from an adjusted linear regression of the association of the SOMAmer with FEV1

P—Obs: P-value from an adjusted linear regression of the association of the SOMAmer with FEV1

FDR P—Obs: False Discovery Rate adjusted p-value from an adjusted linear regression of the association of the SOMAmer with FEV1

**Fig. 4:**
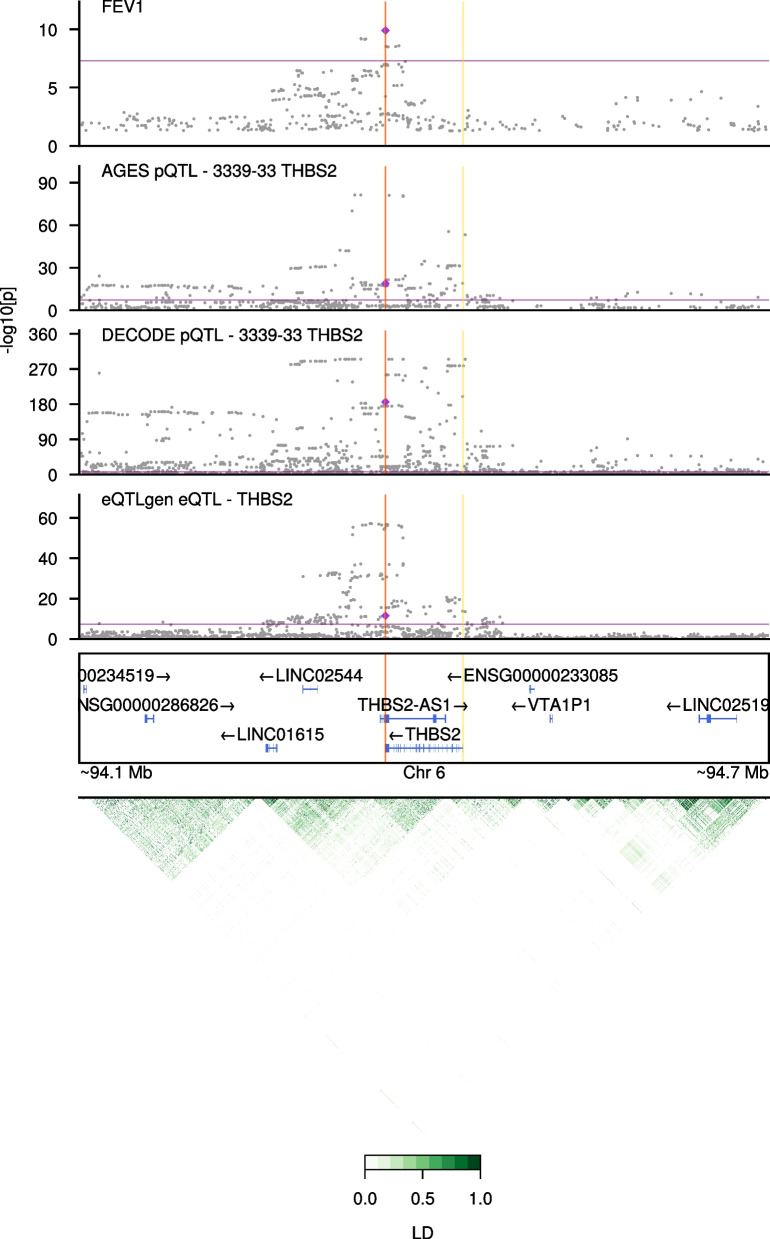
Colocalization plot for Thrombospondin-2 (THBS2). Association results for variants in the *THBS2* region are shown for FEV1 [29], serum THBS2 protein levels in AGES-Reykjavik [24], and plasma THBS2 protein levels in the deCODE study [32] are shown from top to bottom. Grey circles denote individual SNPs from each study. Both purple diamond and red vertical line represent the lead variant (rs3253, 3’ UTR variant). X-axis is genomic position within chromosome 6 and y-axis is -log10 transformed P-values. Purple horizontal line delineates a genome-wide significance threshold at 5 × 10^–8^ and yellow vertical lines represent the gene boundary for *THBS2*. Visualization is restricted to 150,000 bp upstream and downstream of THBS2. Linkage disequilibrium (LD) within the region is plotted at the bottom in green

The 35 proteins representing the 35 SOMAmers identified from the MR analysis (unadjusted p < 0.05; Table 3) were further examined for colocalization evidence between genetic associations for protein levels [24, 32] and FEV1 [29]. Prioritizing the 8 proteins with significant (FDR < 0.05) MR association, we found strong colocalization support (RCP > 0.9) for a single protein, THBS2 (Table 4, Fig. 4, Additional file 5: Table S8). The genetic cis-signal for THBS2 protein expression in AGES-Reykjavik (rs3253 β = − 0.18, p = 1.4 × 10^–19^) colocalized with a signal for FEV1, with the 3’ UTR variant rs3253 being the strongest shared variant (RCP = 0.9976). The THBS2 protein association for this variant replicated in the deCODE cohort (p = 2.1 × 10^–186^, β = − 0.26) (Table 4, Fig. 4). Among the remaining proteins with only nominal associations (p < 0.05) in the MR analysis, TNFSF12 had colocalization support where an upstream variant (rs4968200) associated with TNFSF12 protein levels in AGES-Reykjavik (β = 0.63, p = 5.6 × 10^–133^) colocalized with a signal for FEV1 (RCP = 0.9995; Additional file 3: Figure S3). The other proteins had colocalization probability less than 80%.

**Table 4:** Statistics for proteins with strong colocalization support (regional colocalization probability, RCP > 0.9) from all 35 proteins with nominally significant (p < 0.05) association in the MR analysis

Entrez gene symbol	SNP	RCP (FEV1 × AGES-Reykjavik)	FEV1 GWAS P-value [β]	AGES-Reykjavik protein association p-value [β]	deCODE protein association p-value [β]
THBS2	rs3253^1^	0.9976	1.3 × 10^–10^ [0.02]	1.4 × 10^–19^ [− 0.18]	2.1 × 10^–186^ [− 0.26]
THBS2	rs7756742^1^	0.9775	6.3 × 10^–10^ [0.02]	2.8 × 10^–19^ [− 0.19]	–^3^
THBS2	rs7771838^1^	0.9710	7.0 × 10^–10^ [0.02]	1.8 × 10^–19^ [− 0.19]	–^3^
TNFSF12	rs4968200^2^	0.9995	4.5 × 10^–11^ [0.02]	5.6 × 10^–113^ [0.63]	–^3^

For each protein, all variants with RCP > 0.9 are shown, together with their P-values and beta estimates for associations with FEV1 in a recent GWAS [29] and protein levels in the AGES-Reykjavik [24] and deCODE [32] studies

SNP: Single nucleotide polymorphism, RCP: Regional Colocalization Probability, AGES: Age/Gene Environment Susceptibility, GWAS: Genome-Wide Association Study, THBS2: Thrombospondin-2, TNFSF12: Tumor necrosis factor ligand superfamily member 12, FEV1: Forced Expiratory Volume in 1 s

^1^Lead variant in FEV1 GWAS is rs3253

^2^Lead variant in FEV1 GWAS is rs4968200

^3^Variant data was not available in deCODE summary statistics

Finally, all 530 SOMAmers observationally associated with FEV1 were included in a MR analysis in the reverse direction, i.e., to evaluate if the changes in SOMAmer levels are downstream of changes in lung function. FEV1 was not causally associated with levels of any SOMAmers after adjustment for multiple testing. Data for the 25 SOMAmers that FEV1 was nominally associated with (p < 0.05) in the reverse-MR analysis are shown in Additional file 6: Table S9.

## Discussion

We present findings from a proteomic analysis of pulmonary function with more candidate protein analytes than previously published to our knowledge [11], highlighting several proteins as strong markers of FEV1. Stratification by smoking shows that most associations are driven by ever-smokers. Mendelian randomisation was systematically applied to the candidate markers, revealing proteins whose levels may have a causal effect on lung function. Of those, probabilistic colocalization supported a role of THBS2 and TNFSF12 in affecting lung function. Reverse causation analyses failed to demonstrate that protein level changes associated with FEV1 occur downstream of the phenotype change, although this could partly be due to insufficient power.

Eight proteins were suggested to be causally implicated in lung function based on the MR analysis. However, only three (THBS2, ERO1B and APOM) had consistent direction of effect for the observational and causal estimates. Such discrepancy has been observed when comparing causal and observational estimates [39, 40] for serum proteins. Based on probabilistic quantification, a 3’ UTR variant within *THBS2* was identified as a putative causal variant affecting FEV1 and this lead variant colocalized with THBS2 protein expression in AGES-Reykjavik. The effect of this variant was replicated in an independent cohort for THBS2 protein levels. Because directions of effects were consistent across the datasets and THBS2 had non-revertible causal association to FEV1, supported by colocalization, THBS2 may have biological importance in impaired lung function in the elderly, and could represent a therapeutic target for some forms of respiratory disease. THBS2 is an extracellular matrix protein that has been implicated in various cardiovascular disorders and is also a candidate biomarker for non-small cell lung cancer [41, 42]. THBS2 is involved in tissue repair and interacts with many different ligands in the extracellular matrix, among them matrix metalloproteases and elastase [43]. Although mechanism of THBS2 needs further experimental validation, it is possible that protein levels of THBS2 may influence lung function via extracellular matrix and regenerative pathways. Meanwhile, ERO1B is a disulfide oxidase in the endoplasmic reticulum that is shown to predict survival in pancreatic and pulmonary cancers [44–46] and APOM is an apolipoprotein that is mainly a component of high density lipoproteins and has been associated with COPD severity [47]. Genetic variants flanking the *APOM* gene have been associated with obstructive spirometry measurements [48]. However, it must be kept in mind that the causal association of APOM in our study is based on a single SNP (rs2736163, intronic to *PRRC2A*), thus complicating the interpretation of the MR results.

Despite not reaching the study threshold for statistical significance, some proteins that were nominally associated with lung function in the MR analysis are of interest. First, alpha-1-antitrypsin (SERPINA1) is the best-known protein known to cause COPD as severe deficiency of alpha-1-antitrypsin results in obstructive lung disease [38]. However, in our data, serum levels of SERPINA1 are inversely associated with FEV1 in both observational and MR analyses, contrary to what would possibly be expected, although this directionality is known from previous observational analyses of FEV1 and explained by alpha-1-antitrypsin’s role as an acute phase reactant [49]. Also, polymorphisms that cause mild or intermediate alpha-1-antitrypsin deficiency are not consistently associated with decreased lung function, suggesting that levels of the protein may only affect lung function below a threshold level [50]. Second, TNFSF12 was a protein with a colocalizing pQTL variant with the FEV1 GWAS (Table 4, Fig. 4). TNFSF12 is a member of the Tumor Necrosis Factor (TNF) superfamily, of which one key cytokine, TNF-⍺, is a well-known protein whose levels are disrupted in COPD patients [51]. Presented here are MR based causal evidence and probabilistic colocalization findings that suggest that diseases that present with impaired lung function could be impacted by TNF-⍺ associated pathobiology via TNFSF12. Notable other nominally significant or directionally inconsistent proteins in the findings are matrix metalloproteinase 8 (MMP-8), one of the proteases observationally associated with lung function and implicated in the pathogenesis of COPD [52, 53], TIMP4, an inhibitor of matrix metalloproteinases that has been shown to be upregulated in COPD patients [54] and CD14, levels of which have been shown to be elevated in lungs of smokers [55]. While only eight proteins were significantly associated with FEV1 in MR analyses after multiple testing, FEV1 was not associated with any proteins in analyses in the reverse direction. The data were therefore unable to support prior causal analyses involving inflammatory markers that have suggested reverse causality of FEV1 with inflammatory markers [18] (Additional file 6: Table S7).

The study reveals novel markers with strong observational relations to lung function such as RSPO4, a signalling molecule that is part of the Wnt signalling pathway [56], the tumor marker ALPPL2 [57], the adipokine chemerin (RARRES2), and SVEP1, a protein that is thought to play a role in inflammation in atherosclerosis [58]. In addition, the findings validate the observational associations of some of the previously suggested protein markers of FEV1, such as SFTPD, fibrinogen, IL6, eotaxin and CRP (Additional file 6: Table S2) [13], while the associations of other previously suggested biomarkers of FEV1 such as AGER were not corroborated in this study [13, 59]. Secondary analyses showed that most SOMAmers with the strongest associations with FEV1 were also associated with FEV1/FVC and/or FEV1/FVC under 0.7 in a directionally consistent manner (Additional file 1: Figure S1 and Additional file 6: Table S4). However, under half of all 530 FEV1-associated SOMAmers had statistically significant (FDR < 0.05) associations with the secondary outcomes (Additional file 5: Table S1). Also notable is the finding that among the eight SOMAmers with support for a causal effect on FEV1 from MR analyses (FDR < 0.05), three (RSPO2, TIMP4 and CD14) were observationally associated with FEV1/FVC and/or FEV1/FVC under 0.7, while the remaining five (APOM, THBS2, ERO1B, HDGF, IL1RN) were not (Additional file 5: Table S1). Collectively, these results from secondary analyses suggest that some SOMAmer associations with FEV1 may be explained by other disease mechanisms than obstructive pulmonary disease, such as lung aging. Finally, our findings show that proteins that take part in immune responses, peptidase regulation and extracellular matrix modulation are over-represented among the proteins related to FEV1.

This work is subject to a number of limitations. First, the study is based on SOMAmer technology, a relatively novel aptamer-based technology for protein measurements. While many of these SOMAmers have been validated with encouraging results, it has been pointed out that a minority of SOMAmers could be subject to cross-reactivity with related or homologous proteins [20]. Second, both the AGES-Reykjavik cohort and the UKBiobank and SpiroMeta Consortium are of European ancestry [29]. Therefore, the observed associations could not be generalizable to other populations. Additionally, the AGES-Reykjavik cohort is older than the UKBiobank and SpiroMeta Consortium which could distort comparisons between observational and genetic findings. Third, the processes which mediate the association of SOMAmers with lung function cannot be elucidated from these results. The measure of lung function used in this paper, FEV1, is disproportionally impaired in obstructive lung disease such as COPD. Still, less than half of the FEV1-associated SOMAmers were associated with spirometric obstruction, as discussed above. So, while some associations of SOMAmers with FEV1 may reflect obstructive lung disease, this is likely not the case for all associated SOMAmers. Fourth, subtle differences in genetic structure and datasets between AGES-Reykjavik, UKBiobank, and SpiroMeta cohorts are present and may be contributing to the lack of colocalization for some of the MR identified proteins. For instance, very few MR instruments overlapped with 95% credible-sets identified (Additional file 5: Table S8). In comparisons to UKBiobank, missing variants in the AGES-Reykjavik cohort may contribute to colocalization false negatives. Lastly, many causal estimates are directionally inconsistent with observational estimates. While this phenomenon is known from previous proteogenomic studies, its reasons are unclear.

## Conclusions

In conclusion, this proteogenomic analysis reveals several proteins that are potentially causally related to lung function, most notably THBS2, ERO1B and APOM.

### Supplementary Information

**Additional file 1:** Protein associations with FEV1 (linear regression) compared with (**A**) continuous FEV1/FVC (linear regression) and (**B**) FEV1/FVC under 0.70 (logistic regression).

**Additional file 2:** Over-representation analyses of Gene Ontology (GO) terms associated with genes annotated to FEV1-associated SOMAmers.

**Additional file 3:** Colocalization plot for TNFSF12 protein levels and FEV1.

**Additional file 4:** The results of a leave-one-out analysis for the seven proteins that had significant (FDR < 0.05) causal estimates for FEV1 in the MR analysis and had three or more SNPs as instruments (**A**: THBS2;** B**: ILRN;** C**: TIMP4;** D**: ERO1B;** E**: RSPO2;** F**: HDGF;** G**: CD14). The original causal estimate is shown in red. Each remaining x- and y-axis pair represents a causal estimate and its standard error evaluated without the listed SNP.

**Additional file 5:** Supplementary Tables 1, 5, 6 and 8.

**Additional file 6:** Supplementary Material, including Supplementary Tables 2-4, 7 and 9, as well as Supplementary Table and Figure legends.

## Data Availability

While study materials are not publicly available due to participant privacy, further results or data generated in this study are available upon reasonable request to the authors.
