# Supplementary figures and images for: Proteomic associations with forced expiratory volume: a Mendelian randomisation study
Source: Respir Res. 2024 Jan 18;25:44. doi: 10.1186/s12931-023-02587-z (PMC10797790; doi:10.1186/s12931-023-02587-z)

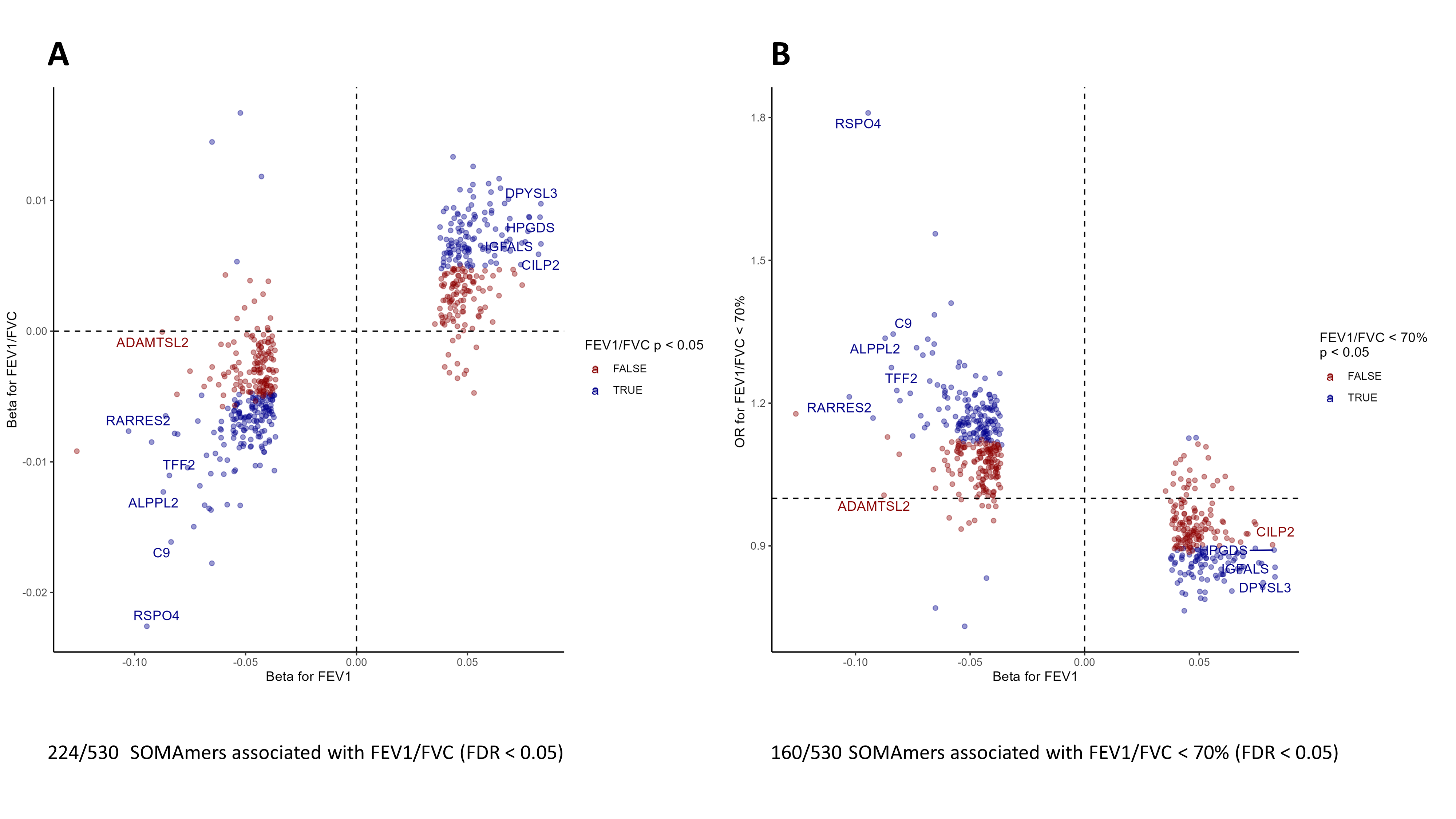

Supplement: Supplementary file 1 — Additional file 1: Protein associations with FEV1 (linear regression) compared with (A) continuous FEV1/FVC (linear regression) and (B) FEV1/FVC under 0.70 (logistic regression). [file 12931_2023_2587_MOESM1_ESM.png]

Dotplot

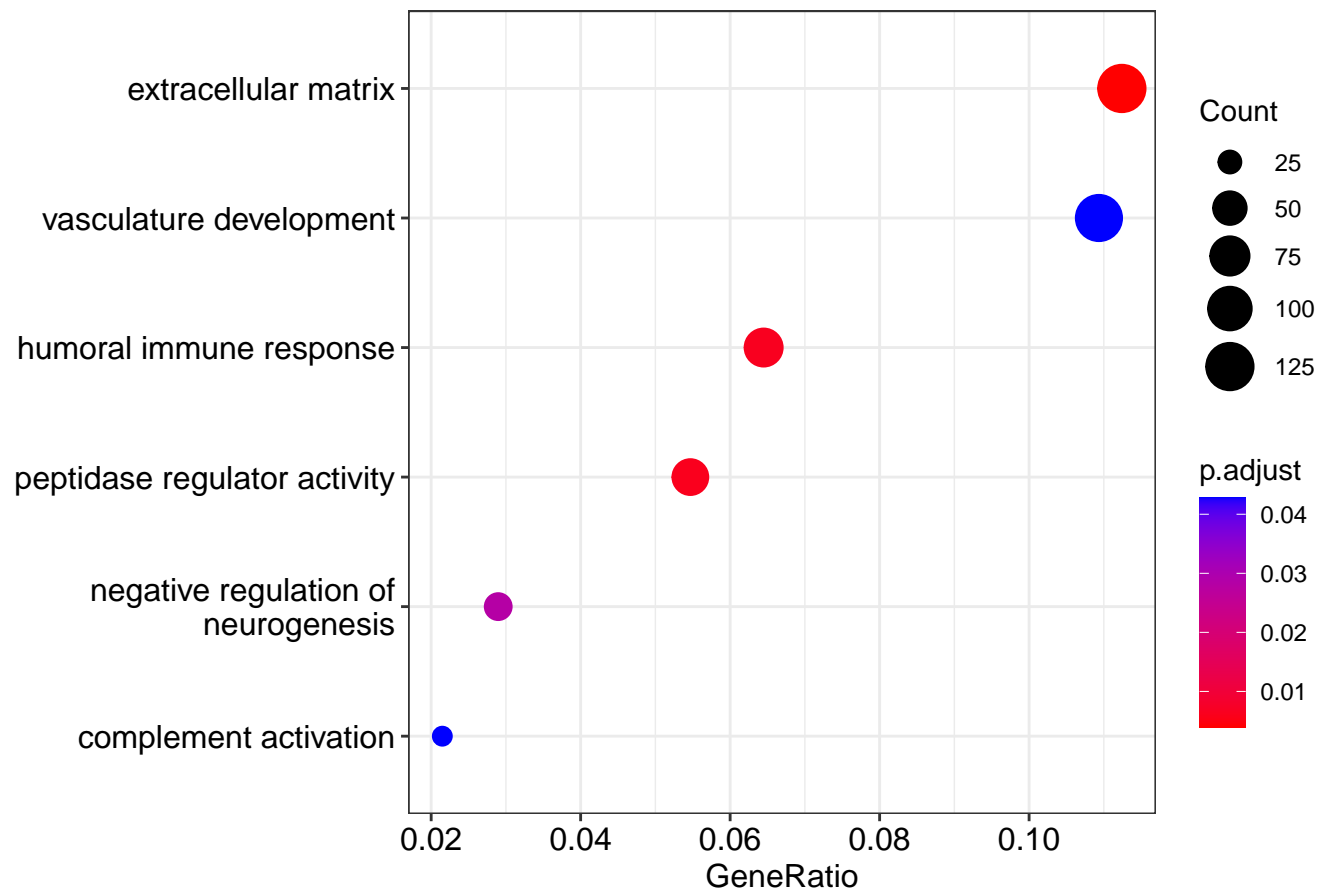

Supplement: Supplementary file 2 — Additional file 2: Over-representation analyses of Gene Ontology (GO) terms associated with genes annotated to FEV1-associated SOMAmers. [file 12931_2023_2587_MOESM2_ESM.pdf]

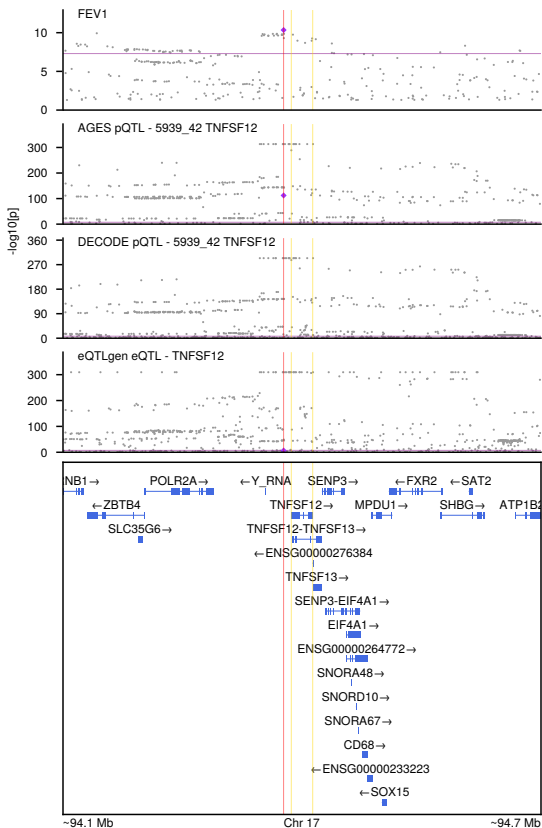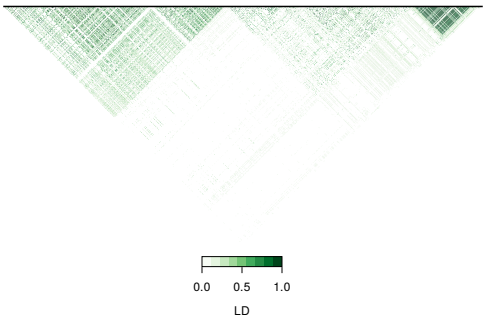

Supplement: Supplementary file 3 — Additional file 3: Colocalization plot for TNFSF12 protein levels and FEV1. [file 12931_2023_2587_MOESM3_ESM.pdf]

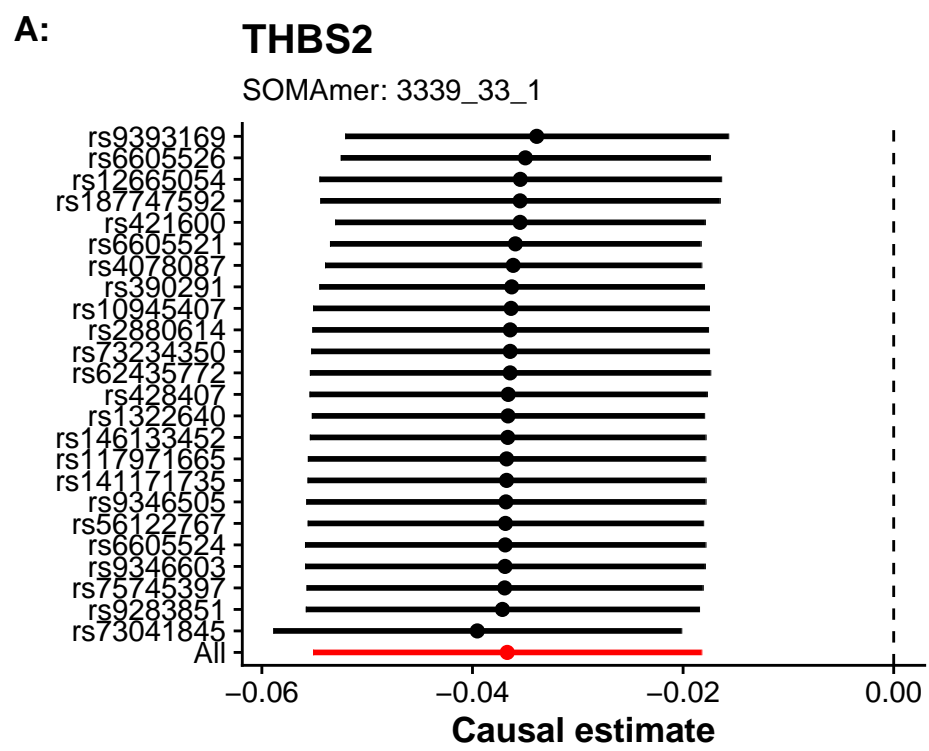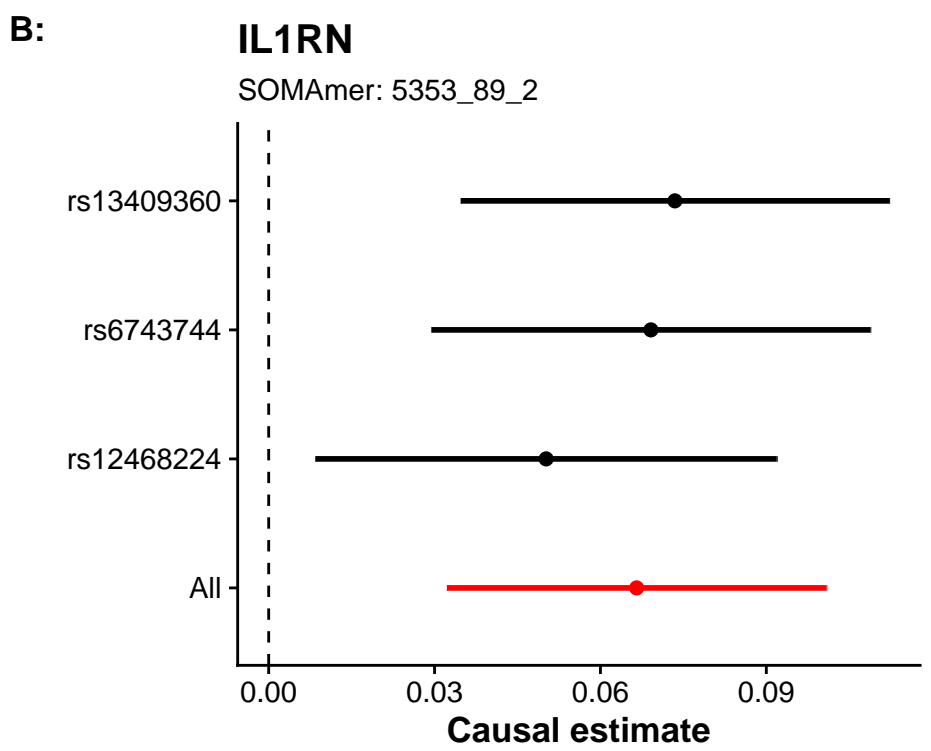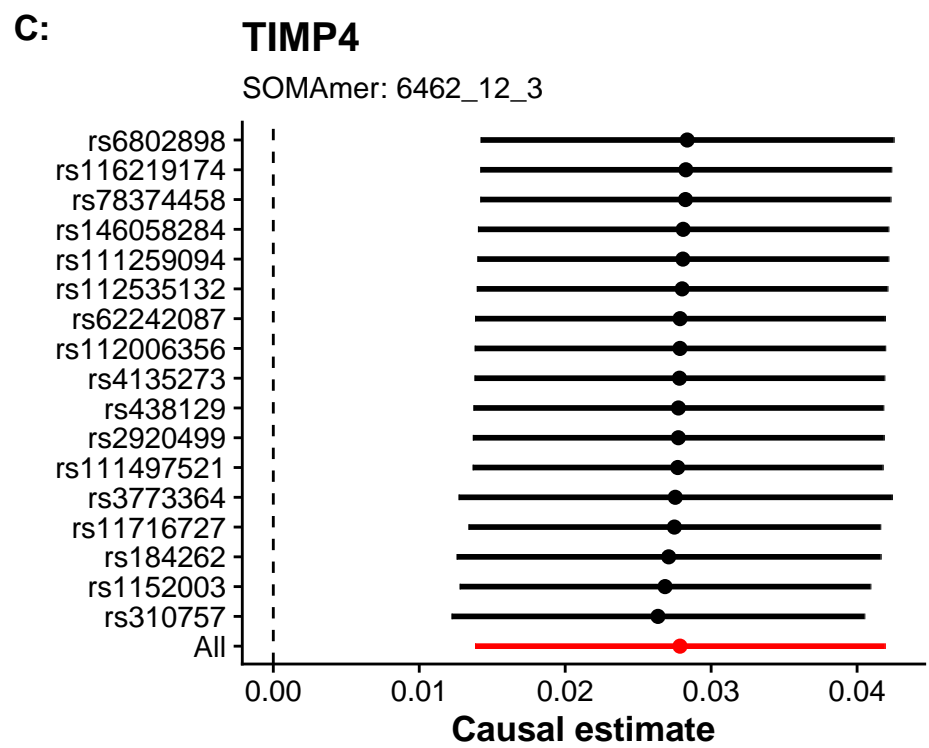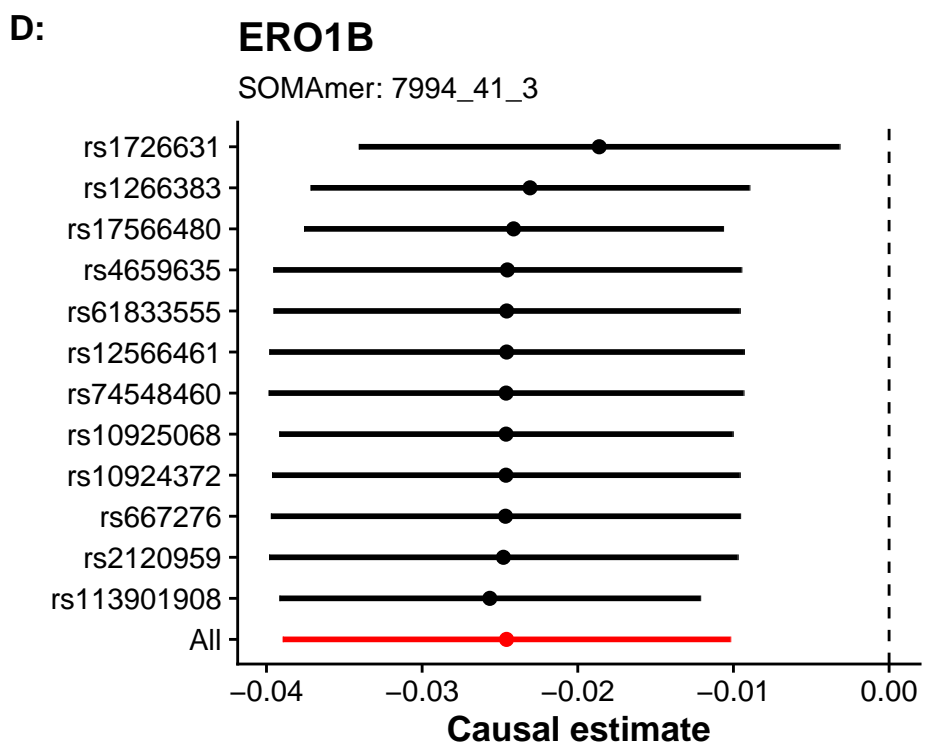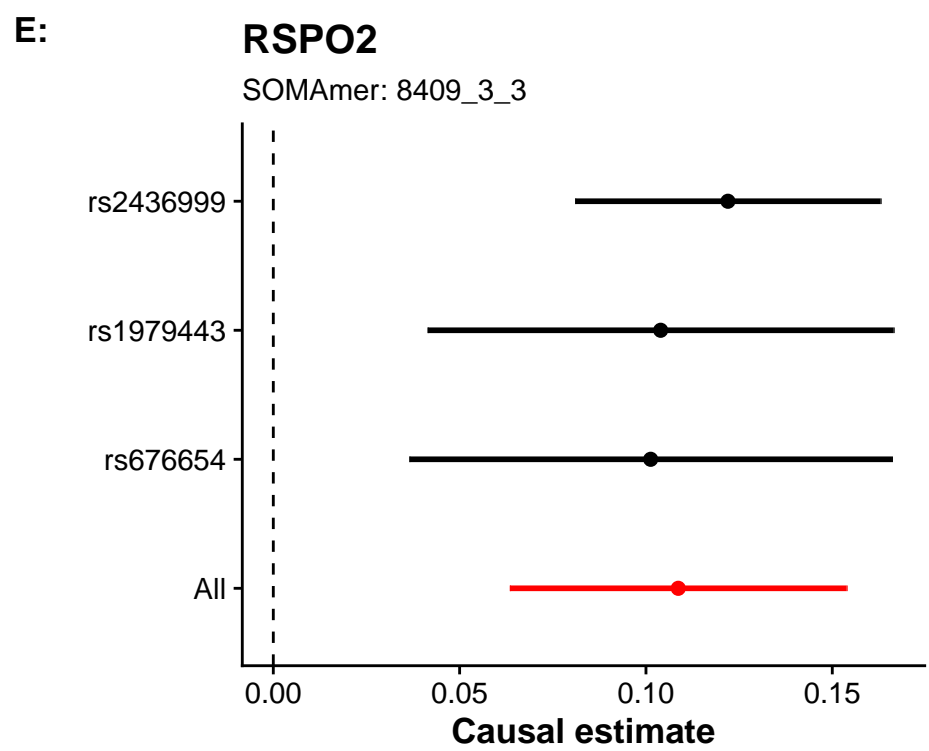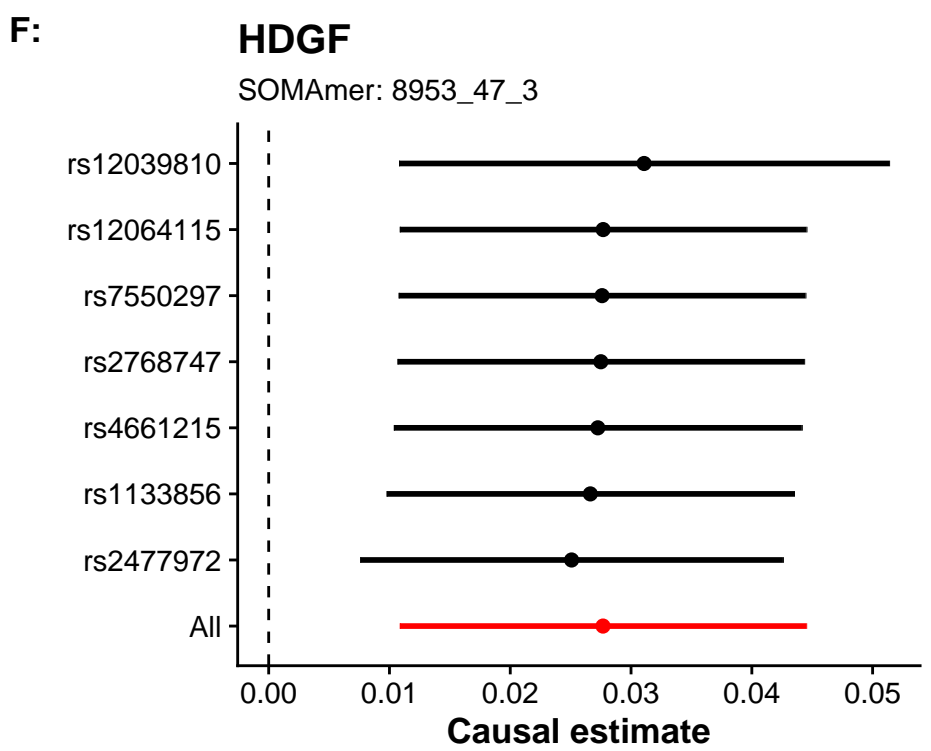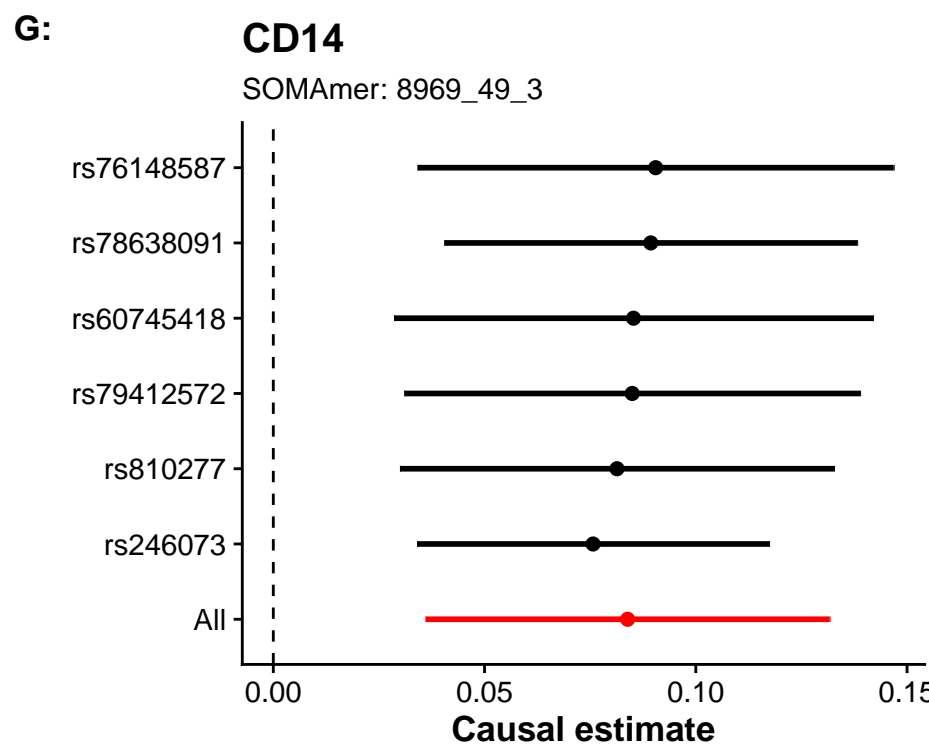

Supplement: Supplementary file 4 — Additional file 4: The results of a leave-one-out analysis for the seven proteins that had significant (FDR < 0.05) causal estimates for FEV1 in the MR analysis and had three or more SNPs as instruments (A: THBS2; B: ILRN; C: TIMP4; D: ERO1B; E: RSPO2; F: HDGF; G: CD14). The original causal estimate is shown in red. Each remaining x- and y-axis pair represents a causal estimate and its standard error evaluated without the listed SNP. [file 12931_2023_2587_MOESM4_ESM.pdf]
