# Supplementary material for: Proteomic associations with forced expiratory volume: a Mendelian randomisation study
Source: Respir Res. 2024 Jan 18;25:44. doi: 10.1186/s12931-023-02587-z (PMC10797790; doi:10.1186/s12931-023-02587-z)
Supplement: Supplementary file 6 — Additional file 6: Supplementary Material, including Supplementary Tables 2-4, 7 and 9, as well as Supplementary Table and Figure legends. [file 12931_2023_2587_MOESM6_ESM.docx]

# **Proteomic associations with forced expiratory volume – a Mendelian randomisation study - Supplementary Material**

Gisli Thor Axelsson^1,2^, Thorarinn Jonmundsson^1^, Youngjae Woo^3^, Elisabet Alexandra Frick^1^, Thor Aspelund^1,4^, Joseph J. Loureiro^3^, Anthony P. Orth^5^, Lori L. Jennings^3^, Gunnar Gudmundsson^4,6^, Valur Emilsson^1,4^, Valborg Gudmundsdottir^1,4^, and Vilmundur Gudnason^1,4^

1: Icelandic Heart Association, Holtasmari 1, 201 Kopavogur, Iceland

2: Landspitali University Hospital, Department of Internal Medicine, 101 Reykjavik, Iceland

3: Novartis Biomedical Research, Cambridge, MA 02139, USA

4: University of Iceland, Faculty of Medicine, 101 Reykjavik, Iceland

5: Novartis Biomedical Research, San Diego, CA 92121, USA
6: Landspitali University Hospital, Department of Respiratory Medicine and Sleep, 108 Reykjavik, Iceland

**Supplementary Tables**

**Table S1** - Observational associations of proteins with FEV1 in AGES-Reykjavik (online Excel file)

**Table S2** – Observational associations of previously suggested biomarkers of FEV1 with FEV1 in AGES-Reykjavik.

| **Protein** | **EGS** | **SOMAmer** | **B** | **P** | **FDR P** | **Cons** | **Citation** |
| --- | --- | --- | --- | --- | --- | --- | --- |
| GRP78 | HSPA5 | - | - | - | - | - | (59) |
| sCD163 | CD163 | 5028_59 | 0.001 | 0.96 | 0.98 | * | (59) |
| CC16 | SCGB1A1 | 10569_28 | -0.034 | 0.02 | 0.12 | Yes | (13) |
| SP-D | SFTPD | 4414_69 | -0.041 | 1.67×10^-3^ | 0.02 | Yes | (13) |
| sRAGE | AGER | 4125_52 | -0.006 | 0.65 | 0.82 | * | (13) |
| CRP | CRP | 4337_49 | -0.065 | 2.7×10^-7^ | 4.9×10^-5^ | Yes | (13) |
| Fibrinogen | FGA | 4907_56 | -0.041 | 2.8×10^-3^ | 0.031 | Yes | (13) |
| Fibrinogen | FGA | 2796_62 | 0.003 | 0.85 | 0.93 | * | (13) |
| IL-6 | IL6 | 4673_13 | -0.047 | 3.8×10^-4^ | 0.0085 | Yes | (60) (61) |
| IL-6 | IL6 | 2573_20 | -0.054 | 1.5×10^-4^ | 0.0046 | Yes | (60) (61) |
| P-selectin | SELP | 4154_57 | -0.004 | 0.77 | 0.89 | * | (60) |
| Eotaxin | CCL11 | 5301_7 | -0.051 | 1.1×10^-4^ | 0.0036 | Yes | (61) |
| IFN-γ | IFNG | 2989_17 | -0.006 | 0.72 | 0.86 | * | (61) |
| IFN-γ | IFNG | 14147_50 | -0.005 | 0.74 | 0.87 | * | (61) |
| IL-10 | IL10 | 2773_50 | 0.004 | 0.78 | 0.90 | * | (61) |
| IL-10 | IL10 | 13723_6 | 0.02 | 0.17 | 0.41 | * | (61) |
| IL-2 | IL2 | 3070_1 | 0.017 | 0.23 | 0.47 | * | (61) |
| IL-8 | CXCL8 | 3447_64 | 0.004 | 0.77 | 0.89 | * | (61) |
| TNF-α | TNF | 5936_53 | -0.023 | 0.08 | 0.27 | * | (61) |
| TNF-α | TNF | 5692_79 | -0.028 | 0.04 | 0.17 | Yes | (61) |

Adjusted for sex, age, age squared, height and height squared.

-: No data available
*: Not applicable as observational analysis in AGES-Reykjavik were not significant
Protein: Protein name
EGS: Entrez Gene symbol
SOMAmer: SOMAmer number
95% CI: 95% Confidence interval
P: P-value
FDR P: False Discovery Rate adjusted p-value
Cons: Consistency of direction of β with previous data

**Table S3** – Observational associations of proteins with FEV_1_ stratified by ever-smoking

|  |  | **EVER-SMOKERS**  **(n = 863)** | | | **NEVER-SMOKERS**  **(n = 578)** | | |
| --- | --- | --- | --- | --- | --- | --- | --- |
| **SOMA** | **EGS** | **β** | **95% CI** | **P** | **β** | **95% CI** | **P** |
| 3079_62_2 | RARRES2 | -0.12 | -0.16 - -0.08 | 5.85×10^-9^ | -0.032 | -0.073 - 0.008 | 0.119 |
| 8464_31_3 | RSPO4 | -0.095 | -0.133 - -0.057 | 9.61×10^-7^ | -0.029 | -0.068 - 0.011 | 0.152 |
| 7813_6_3 | ALPPL2 | -0.097 | -0.132 - -0.061 | 1.05×10^-7^ | -0.002 | -0.043 - 0.04 | 0.932 |
| 2292_17_4 | C9 | -0.096 | -0.131 - -0.06 | 2.05×10^-7^ | -0.038 | -0.074 - -0.002 | 0.038 |
| 12549_33_3 | HPGDS | 0.092 | 0.056 - 0.129 | 8.58×10^-7^ | 0.053 | 0.02 - 0.087 | 2.05×10^-3^ |
| 6605_17_3 | IGFALS | 0.088 | 0.052 - 0.125 | 2.70×10^-6^ | 0.046 | 0.011 - 0.081 | 0.01 |
| 12707_26_3 | DPYSL3 | 0.098 | 0.062 - 0.135 | 1.91×10^-7^ | 0.027 | -0.009 - 0.062 | 0.14 |
| 9191_8_3 | TFF2 | -0.104 | -0.141 - -0.067 | 3.37×10^-8^ | -0.011 | -0.049 - 0.027 | 0.573 |
| 8841_65_3 | CILP2 | 0.095 | 0.059 - 0.131 | 3.11×10^-7^ | 0.042 | 0.006 - 0.077 | 0.022 |
| 6379_62_3 | ADAMTSL2 | -0.079 | -0.119 - -0.039 | 1.00×10^-4^ | -0.068 | -0.107 - -0.029 | 6.25×10^-4^ |
| 11178_21_3 | SVEP1 | -0.082 | -0.121 - -0.044 | 3.43×10^-5^ | -0.082 | -0.12 - -0.045 | 1.64×10^-5^ |
| 2677_1_1 | EGFR | 0.099 | 0.064 - 0.135 | 6.58×10^-8^ | 0.027 | -0.006 - 0.061 | 0.11 |
| 8885_6_3 | CACNA2D3 | 0.10 | 0.063 - 0.137 | 1.10×10^-7^ | 0.007 | -0.029 - 0.043 | 0.704 |
| 11109_56_3 | SVEP1 | -0.082 | -0.12 - -0.043 | 3.25×10^-5^ | -0.081 | -0.119 - -0.043 | 2.86×10^-5^ |
| 13722_105_3 | C9 | -0.081 | -0.116 - -0.046 | 7.35×10^-6^ | -0.037 | -0.073 - -0.001 | 0.044 |
| 10514_5_3 | PTGDS | -0.098 | -0.14 - -0.057 | 3.98×10^-6^ | -0.042 | -0.083 - -0.001 | 0.044 |
| 2658_27_1 | NTRK3 | 0.107 | 0.07 - 0.144 | 2.47×10^-8^ | 0.008 | -0.027 - 0.043 | 0.652 |
| 6390_18_3 | NPS | 0.084 | 0.045 - 0.123 | 2.33×10^-5^ | 0.063 | 0.027 - 0.10 | 7.91×10^-4^ |
| 6075_61_3 | HEXB | 0.079 | 0.04 - 0.118 | 7.27×10^-5^ | 0.057 | 0.021 - 0.092 | 1.86×10^-3^ |
| 3396_54_2 | REN | -0.09 | -0.127 - -0.052 | 3.41×10^-6^ | -0.019 | -0.056 - 0.018 | 0.317 |
| 8323_163_3 | TFF3 | -0.094 | -0.133 - -0.055 | 2.95×10^-6^ | -0.023 | -0.06 - 0.013 | 0.209 |
| 2609_59_2 | CST3 | -0.09 | -0.131 - -0.05 | 1.14×10^-5^ | -0.042 | -0.084 – 0.0 | 0.049 |
| 3216_2_2 | PIGR | -0.062 | -0.098 - -0.027 | 6.67×10^-4^ | -0.03 | -0.067 - 0.007 | 0.111 |
| 4496_60_2 | MMP12 | -0.075 | -0.112 - -0.038 | 7.69×10^-5^ | -0.021 | -0.058 - 0.016 | 0.261 |
| 5632_6_3 | CRTAC1 | 0.079 | 0.039 - 0.118 | 9.01×10^-5^ | 0.038 | 0.001 - 0.075 | 0.045 |

Results are shown for the 25 proteins with the most significant associations with FEV1 in the whole cohort.

**Table S4** – Results from regression models of FEV1/FVC ratio for the 25 proteins with the most significant associations for FEV1 as selected by the lowest P-values

| **SOMA** | **EGS** | **FEV1 β** | **FEV1 CI** | **FEV1 P** | **FEV1 FDR** | **FEV1/FVC β** | **FEV1/FVC CI** | **FEV1/FVC P** | **FEV1/FVC FDR** | **FEV1/FVC under 0.70 OR** | | **FEV1/FVC under 0.70 OR** | | **FEV1/FVC under 0.70 P** | | **FEV1/FVC under 0.70 FDR** | |
| --- | --- | --- | --- | --- | --- | --- | --- | --- | --- | --- | --- | --- | --- | --- | --- | --- | --- |
| 3079_62_2 | RARRES2 | -0.103 | -0.132 - -0.074 | 7.76×10^-12^ | 3.71×10^-8^ | -0.008 | -0.013 - -0.002 | 5.26×10^-3^ | 0.021 | 1.213 | | 1.07 - 1.375 | | 2.50×10^-3^ | | 0.018 | |
| 8464_31_3 | RSPO4 | -0.094 | -0.122 - -0.067 | 1.86×10^-11^ | 4.44×10^-8^ | -0.023 | -0.027 - -0.018 | 5.74×10^-19^ | 3.04×10^-16^ | 1.81 | | 1.594 - 2.055 | | 6.02×10^-20^ | | 3.19×10^-17^ | |
| 7813_6_3 | ALPPL2 | -0.087 | -0.113 - -0.061 | 1.23×10^-10^ | 1.96×10^-7^ | -0.012 | -0.017 - -0.008 | 5.76×10^-7^ | 2.18×10^-5^ | 1.336 | | 1.192 - 1.498 | | 6.98×10^-7^ | | 6.17×10^-5^ | |
| 2292_17_4 | C9 | -0.084 | -0.109 - -0.058 | 3.04×10^-10^ | 3.63×10^-7^ | -0.016 | -0.021 - -0.011 | 2.05×10^-11^ | 5.42×10^-9^ | 1.345 | | 1.201 - 1.507 | | 3.21×10^-7^ | | 3.41×10^-5^ | |
| 12549_33_3 | HPGDS | 0.083 | 0.057 - 0.109 | 5.12×10^-10^ | 4.37×10^-7^ | 0.009 | 0.004 - 0.013 | 3.23×10^-4^ | 3.00×10^-3^ | 0.891 | | 0.798 - 0.996 | | 0.042 | | 0.099 | |
| 6605_17_3 | IGFALS | 0.083 | 0.057 - 0.109 | 5.99×10^-10^ | 4.37×10^-7^ | 0.007 | 0.002 - 0.011 | 6.40×10^-3^ | 0.023 | 0.855 | | 0.765 - 0.956 | | 5.72×10^-3^ | | 0.028 | |
| 12707_26_3 | DPYSL3 | 0.083 | 0.057 - 0.109 | 6.40×10^-10^ | 4.37×10^-7^ | 0.01 | 0.005 - 0.015 | 6.70×10^-5^ | 9.35×10^-4^ | 0.835 | | 0.746 - 0.934 | | 1.64×10^-3^ | | 0.014 | |
| 9191_8_3 | TFF2 | -0.084 | -0.111 - -0.058 | 7.42×10^-10^ | 4.44×10^-7^ | -0.011 | -0.016 - -0.006 | 9.34×10^-6^ | 2.25×10^-4^ | 1.275 | | 1.136 - 1.43 | | 3.63×10^-5^ | | 1.22×10^-3^ | |
| 8841_65_3 | CILP2 | 0.082 | 0.056 - 0.108 | 1.09×10^-9^ | 5.78×10^-7^ | 0.006 | 0.001 - 0.011 | 0.017 | 0.042 | 0.902 | | 0.807 - 1.009 | | 0.071 | | 0.136 | |
| 6379_62_3 | ADAMTSL2 | -0.088 | -0.116 - -0.059 | 3.18×10^-9^ | 1.52×10^-6^ | 0 | -0.005 - 0.005 | 0.982 | 0.984 | 1.007 | | 0.891 - 1.138 | | 0.917 | | 0.929 | |
| 11178_21_3 | SVEP1 | -0.082 | -0.11 - -0.054 | 1.03×10^-8^ | 4.46×10^-6^ | -0.008 | -0.013 - -0.003 | 2.70×10^-3^ | 0.013 | 1.226 | | 1.088 - 1.383 | | 8.48×10^-4^ | | 0.01 | |
| 2677_1_1 | EGFR | 0.075 | 0.049 - 0.1 | 1.21×10^-8^ | 4.56×10^-6^ | 0.007 | 0.002 - 0.011 | 4.65×10^-3^ | 0.019 | 0.895 | | 0.802 - 0.998 | | 0.046 | | 0.103 | |
| 8885_6_3 | CACNA2D3 | 0.077 | 0.051 - 0.104 | 1.24×10^-8^ | 4.56×10^-6^ | 0.008 | 0.003 - 0.012 | 2.00×10^-3^ | 0.011 | 0.863 | | 0.771 - 0.966 | | 0.011 | | 0.041 | |
| 11109_56_3 | SVEP1 | -0.081 | -0.109 - -0.053 | 1.88×10^-8^ | 6.44×10^-6^ | -0.008 | -0.013 - -0.003 | 2.55×10^-3^ | 0.013 | 1.205 | | 1.069 - 1.358 | | 2.25×10^-3^ | | 0.016 | |
| 13722_105_3 | C9 | -0.073 | -0.099 - -0.048 | 2.23×10^-8^ | 7.07×10^-6^ | -0.015 | -0.02 - -0.01 | 3.07×10^-10^ | 5.42×10^-8^ | 1.316 | | 1.177 - 1.472 | | 1.48×10^-6^ | | 1.10×10^-4^ | |
| 10514_5_3 | PTGDS | -0.086 | -0.116 - -0.056 | 2.37×10^-8^ | 7.07×10^-6^ | -0.006 | -0.012 - -0.001 | 0.021 | 0.05 | 1.129 | | 0.995 - 1.281 | | 0.06 | | 0.123 | |
| 2658_27_1 | NTRK3 | 0.075 | 0.048 - 0.101 | 3.67×10^-8^ | 1.03×10^-5^ | 0.004 | -0.001 - 0.008 | 0.154 | 0.214 | 0.946 | | 0.845 - 1.058 | | 0.329 | | 0.417 | |
| 6390_18_3 | NPS | 0.078 | 0.05 - 0.106 | 4.61×10^-8^ | 1.22×10^-5^ | 0.009 | 0.004 - 0.014 | 7.94×10^-4^ | 5.61×10^-3^ | 0.823 | | 0.731 - 0.926 | | 1.24×10^-3^ | | 0.012 | |
| 6075_61_3 | HEXB | 0.076 | 0.049 - 0.103 | 6.78×10^-8^ | 1.71×10^-5^ | 0.007 | 0.002 - 0.012 | 7.71×10^-3^ | 0.026 | 0.864 | | 0.769 - 0.971 | | 0.014 | | 0.048 | |
| 3396_54_2 | REN | -0.075 | -0.102 - -0.048 | 7.42×10^-8^ | 1.77×10^-5^ | -0.003 | -0.008 - 0.002 | 0.229 | 0.292 | 1.131 | | 1.007 - 1.269 | | 0.037 | | 0.09 | |
| 8323_163_3 | TFF3 | -0.076 | -0.104 - -0.048 | 9.06×10^-8^ | 2.06×10^-5^ | -0.01 | -0.016 - -0.005 | 5.50×10^-5^ | 8.33×10^-4^ | 1.221 | | 1.084 - 1.374 | | 9.91×10^-4^ | | 0.011 | |
| 2609_59_2 | CST3 | -0.081 | -0.111 - -0.051 | 1.14×10^-7^ | 2.49×10^-5^ | -0.005 | -0.01 - 0.001 | 0.082 | 0.135 | | 1.093 | | 0.964 - 1.238 | | 0.165 | | 0.249 |
| 3216_2_2 | PIGR | -0.07 | -0.096 - -0.044 | 1.35×10^-7^ | 2.81×10^-5^ | -0.005 | -0.01 - 0 | 0.042 | 0.083 | | 1.148 | | 1.029 - 1.282 | | 0.014 | | 0.048 |
| 4496_60_2 | MMP12 | -0.072 | -0.098 - -0.045 | 1.52×10^-7^ | 3.03×10^-5^ | -0.007 | -0.012 - -0.002 | 5.56×10^-3^ | 0.021 | | 1.173 | | 1.047 - 1.315 | | 5.80×10^-3^ | | 0.028 |
| 5632_6_3 | CRTAC1 | 0.074 | 0.046 - 0.102 | 1.93×10^-7^ | 3.69×10^-5^ | 0.005 | 0 - 0.01 | 0.049 | 0.093 | | 0.95 | | 0.845 - 1.068 | | 0.394 | | 0.472 |

SOMA: SOMAmer number
EGS: Entrez Gene symbol
FEV1 B: Beta estimate from an adjusted linear regression of the association of the SOMAmer with FEV1
FEV1 CI: 95% confidence interval from an adjusted linear regression of the association of the SOMAmer with FEV1
FEV1 P: P-value from an adjusted linear regression of the association of the SOMAmer with FEV1
FEV1 FDR: False Discovery Rate adjusted p-value from an adjusted linear regression of the association of the SOMAmer with FEV1
FEV1/FVC B: Beta estimate from an adjusted linear regression of the association of the SOMAmer with FEV1/FVC
FEV1/FVC CI: 95% confidence interval from an adjusted linear regression of the association of the SOMAmer with FEV1/FVC
FEV1/FVC P: P-value from an adjusted linear regression of the association of the SOMAmer with FEV1/FVC
FEV1/FVC under 0.70 B: Beta estimate from an adjusted logistic regression of the association of the SOMAmer with an outcome defined as FEV1/FVC < 0.70
FEV1/FVC under 0.70 CI: 95% confidence interval from an adjusted logistic regression of the association of the SOMAmer with an outcome defined as FEV1/FVC < 0.70
FEV1/FVC under 0.70 P: P-value from an adjusted logistic regression of the association of the SOMAmer with an outcome defined as FEV1/FVC < 0.70

**Table S5** – Results of over-representation analyses of GO terms related to genes annotated to FEV1 associated SOMAmers (online Excel file)

**Table S6** – Genetic instruments used in MR analyses for SOMAmers significantly associated with FEV_1_ (online Excel file)

**Table S7** – MR associations of proteins previously suggested to be causally related to FEV_1_

| **Protein** | **EGS** | **SOMAmer** | **B** | **P** | **FDR** | **MR B** | **MR P** | **MR FDR** | **Rev MR B** | **Rev MR P** | **Rev MR FDR** | **Citation** |
| --- | --- | --- | --- | --- | --- | --- | --- | --- | --- | --- | --- | --- |
| SP-D | SFTPD | 4414_69 | -0.04 | 0.002 | 0.02 | - | - | - | 0.12 | 0.07 | 0.95 | (13) |
| CRP | CRP | 4337_49 | -0.07 | 2.7×10^-7^ | 4.9×10^-5^ | 0.0002 | 0.99 | 1.0 | -0.09 | 0.13 | 0.95 | (13) |
| Fibrinogen | FGA | 4907_56 | -0.04 | 0.003 | 0.03 | -0.003 | 0.86 | 0.97 | -0.09 | 0.13 | 0.95 | (13) |
| IL-6 | IL6 | 4673_13 | -0.05 | 3.7×10^-4^ | 0.008 | - | - | - | -0.0003 | 1 | 1 | (60) (61) |
| IL-6 | IL6 | 2573_20 | -0.05 | 1.5×10^-4^ | 0.005 | - | - | - | -0.03 | 0.67 | 0.95 | (60) (61) |
| Eotaxin | CCL11 | 5301_7 | -0.05 | 0.0001 | 0.004 | -0.04 | 0.11 | 0.46 | -0.02 | 0.77 | 0.97 | (60) |

-: No data available
Protein: Protein name
EGS: Entrez Gene symbol
SOMAmer: SOMAmer number
B: Beta estimate from an adjusted linear regression of the association of the SOMAmer with FEV1
P: P-value from an adjusted linear regression of the association of the SOMAmer with FEV1
FDR: False Discovery Rate adjusted p-value from an adjusted linear regression of the association of the SOMAmer with FEV1
MR B: Weighted median estimate from the mendelian randomization analysis of the association of the SOMAmer with FEV1
MR P: P-value for the weighted median estimate from the mendelian randomization analysis of the association of the SOMAmer with FEV1
MR FDR: False Discovery Rate adjusted p-value for the weighted median estimate from the mendelian randomization analysis of the association of the SOMAmer with FEV1
FDR P: False Discovery Rate adjusted p-value for the weighted median estimate from the mendelian randomization analysis of the association of the SOMAmer with FEV1
REV MR B: Weighted median estimate from the “reverse” mendelian randomization analysis of the association of FEV1 with the SOMAmer
REV MR P: P-value for the weighted median estimate from the “reverse” mendelian randomization analysis of the association of FEV1 with the SOMAmer
REV MR FDR: False Discovery Rate adjusted p-value for the weighted median estimate from the “reverse” mendelian randomization analysis of the association of FEV1 with the SOMAmer

**Table S8** – Overlap between colocalization credible sets and MR instruments (online Excel file)

**Table S9** – Results of reverse Mendelian randomisation analysis of the association of FEV1 with SOMAmers

| **SOMA** | **EGS** | **nSNP** | **MR B** | **SE** | **MR P** | **MR FDR** | **Obs B** | **Obs B** | **Obs FDR** |
| --- | --- | --- | --- | --- | --- | --- | --- | --- | --- |
| *9282_12_3* | *CRISP2* | *366* | *0.195* | *0.056* | *4.94e-04* | *0.262* | *0.078* | *3.24e-07* | *5.42e-05* |
| *7784_1_3* | *KNG1* | *365* | *-0.166* | *0.054* | *2.21e-03* | *0.585* | *-0.046* | *3.43e-03* | *0.037* |
| 3676_15_3 | IGF2R | 366 | -0.17 | 0.062 | 6.35e-03 | 0.731 | 0.042 | 1.56e-03 | 0.021 |
| *9851_9_3* | *FABP4* | *366* | *-0.135* | *0.053* | *0.011* | *0.731* | *-0.043* | *3.95e-03* | *0.04* |
| *5034_79_1* | *PRSS2* | *366* | *-0.155* | *0.061* | *0.012* | *0.731* | *-0.037* | *5.00e-03* | *0.047* |
| *8484_24_3* | *LEP* | *366* | *-0.132* | *0.053* | *0.013* | *0.731* | *-0.052* | *7.48e-04* | *0.013* |
| 12395_86_3 | DARS2 | 366 | 0.144 | 0.06 | 0.015 | 0.731 | -0.038 | 4.74e-03 | 0.046 |
| *14260_112_3* | *NET1* | *366* | *-0.142* | *0.059* | *0.017* | *0.731* | *-0.044* | *1.30e-03* | *0.019* |
| *7994_41_3* | *ERO1B* | *366* | *-0.142* | *0.061* | *0.02* | *0.731* | *-0.038* | *4.28e-03* | *0.042* |
| *3324_51_1* | *LY9* | *366* | *0.139* | *0.06* | *0.02* | *0.731* | *0.052* | *1.31e-04* | *4.11e-03* |
| 12740_55_3 | FEV | 366 | 0.139 | 0.06 | 0.021 | 0.731 | -0.043 | 1.00e-03 | 0.016 |
| *6940_18_3* | *JPH1* | *366* | *-0.128* | *0.056* | *0.022* | *0.731* | *-0.039* | *5.40e-03* | *0.049* |
| *3505_6_2* | *LTA* | *365* | *0.137* | *0.06* | *0.023* | *0.731* | *0.058* | *1.77e-05* | *1.01e-03* |
| *6380_23_3* | *MRPL58* | *365* | *-0.146* | *0.064* | *0.024* | *0.731* | *-0.055* | *7.32e-05* | *2.84e-03* |
| *2575_5_5* | *LEP* | *366* | *-0.119* | *0.053* | *0.025* | *0.731* | *-0.065* | *3.59e-05* | *1.75e-03* |
| *4891_50_1* | *GCG* | *366* | *-0.135* | *0.061* | *0.026* | *0.731* | *-0.044* | *1.07e-03* | *0.016* |
| *8476_11_3* | *CHGA* | *364* | *-0.131* | *0.059* | *0.026* | *0.731* | *-0.062* | *3.97e-06* | *3.52e-04* |
| *5737_61_3* | *SEMA4D* | *366* | *0.135* | *0.061* | *0.027* | *0.731* | *0.044* | *9.37e-04* | *0.015* |
| *10563_13_3* | *LYSMD3* | *366* | *-0.12* | *0.054* | *0.028* | *0.731* | *-0.045* | *3.20e-03* | *0.035* |
| *2867_52_2* | *AKT1* | *365* | *0.132* | *0.06* | *0.028* | *0.731* | *0.067* | *7.21e-07* | *1.04e-04* |
| *6236_51_3* | *CTHRC1* | *366* | *-0.105* | *0.051* | *0.037* | *0.939* | *-0.053* | *5.04e-04* | *0.01* |
| *2950_57_2* | *IGFBP4* | *366* | *-0.122* | *0.06* | *0.041* | *0.947* | *-0.054* | *1.17e-04* | *3.79e-03* |
| *11178_21_3* | *SVEP1* | *366* | *-0.117* | *0.058* | *0.042* | *0.947* | *-0.082* | *1.03e-08* | *4.46e-06* |
| *13098_93_3* | *VEGFD* | *366* | *-0.112* | *0.055* | *0.044* | *0.947* | *-0.048* | *7.22e-04* | *0.013* |
| *13043_157_3* | *ATAD2* | *366* | *-0.102* | *0.052* | *0.05* | *0.947* | *-0.042* | *5.01e-03* | *0.047* |

Shown are data for observationally significant SOMAmers that had nominally significant associations using Mendelian randomisation. Observational data are adjusted for sex, age, age squared, height and height squared.

*Italic* = Consistent in direction between MR analyses and observational analyses
SOMA: SOMAmer number
EGS: Entrez Gene symbol
nSNP: Number of single nucleotide polymorphisms used in analysis
MR B: Weighted median estimate from the mendelian randomization analysis of the association of FEV1 with the SOMAmer
SE: Standard error from the mendelian randomization analysis of the association of FEV1 with the SOMAmer
MR P: P-value for the weighted median estimate from the mendelian randomization analysis of the association of FEV1 with the SOMAmer
MR FDR: False Discovery Rate adjusted p-value for the weighted median estimate from the mendelian randomization analysis of the association of FEV1 with the SOMAmer
Obs B: Beta estimate from an adjusted linear regression of the association of the SOMAmer with FEV1
Obs P: P-value from an adjusted linear regression of the association of the SOMAmer with FEV1
Obs FDR: False Discovery Rate adjusted p-value from an adjusted linear regression of the association of the SOMAmer with FEV1

**Supplementary Figure legends**

**Figure S1 –** Results of regression models of FEV1 compared with regression models of continuous FEV1/FVC (panel A) and logistic regression models with FEV1/FVC under 0.70 (panel B).

**Figure S2** – Results of over-representation analyses of GO terms associated with genes annotated to FEV1 associated SOMAmers.

**Figure S3** – Colocalization plot for TNFSF12 protein levels and FEV1.

**Figure S4** – The results of a leave-one-out analysis for the seven proteins that had significant (FDR<0.05) causal estimates for FEV1 in the MR analysis and had three or more SNPs as instruments (A: THBS2; B: ILRN; C: TIMP4; D: ERO1B; E: RSPO2; F: HDGF; G:CD14). The original causal estimate is shown in red. Each remaining x- and y-axis pair represents a causal estimate and its standard error evaluated without the listed SNP. None of the MR associations for the proteins examined were dependent on a single variant.

**Supplemental References**

60. Walter RE, Wilk JB, Larson MG, Vasan RS, Keaney JF, Jr., Lipinska I, et al. Systemic inflammation and COPD: the Framingham Heart Study. Chest. 2008;133(1):19-25.

61. Bradford E, Jacobson S, Varasteh J, Comellas AP, Woodruff P, O'Neal W, et al. The value of blood cytokines and chemokines in assessing COPD. Respir Res. 2017;18(1):180.
